# Oxymatrine inhibited the progression of renal cell carcinoma by increasing TOR1AIP1 expression

**DOI:** 10.3389/fphar.2025.1611069

**Published:** 2025-10-02

**Authors:** Xuechuan Yan, Kai Zhao, Zongliang Zhang, Xinbao Yin, Han Yang, Zaiqing Jiang, Tianzhen He, Ke Wang

**Affiliations:** ^1^ Department of Urology, the Affiliated Hospital of Shandong Second Medical University, Weifang, China; ^2^ Department of Urology, The Affiliated Hospital of Qingdao University, Qingdao, Shangdong, China; ^3^ Institute of Pain Medicine and Special Environmental Medicine, Co-innovation Center of Neuroregeneration, Nantong University, Nantong, China

**Keywords:** oxymatrine, TOR1AIP1, renal cell carcinoma, JNK signaling pathway, therapeutic strategy

## Abstract

**Aims:**

The extract of Radix sophorae tonkinensis, known as oxymatrine (OMT), demonstrates anticancer properties. This investigation explored the influence of oxymatrine on renal cell carcinoma (RCC) and elucidated the associated molecular mechanisms, both *in vitro* and *in vivo*.

**Methods:**

RNA-seq was used to evaluate target genes regulated by OMT. The potential target gene TOR1AIP1 was identified, and the expression of TOR1AIP1 was analyzed in RCC cell lines (Caki-1 and 786-O cells) after treatment with OMT. Further functional assays, including *in vitro* proliferation, migration, and invasion experiments, were performed. Additionally, overexpression experiments were used to confirm the role of TOR1AIP1 in RCC. *In vivo* assays using a nude mouse model were conducted to evaluate the effect of OMT on tumor growth.

**Results:**

We identified TOR1AIP1 as the potential target gene ofOMT. Among the tested compounds, OMT significantly increased the expression of TOR1AIP1 in RCC cells. OMT inhibited RCC progression, including cell proliferation, migration, and invasion, by upregulating TOR1AIP1. Mechanistically, overexpression of TOR1AIP1 in RCC cells markedly inactivated the JNK signaling pathway and suppressed RCC development. *In vivo*, OMT treatment significantly inhibited tumor growth, consistent with the *in vitro* findings.

**Conclusion:**

Our findings demonstrate that OMT suppresses RCC progression by increasing the expression of TOR1AIP1 and inactivating the JNK signaling pathway. These findings support TOR1AIP1 as a mechanistic mediator of OMT’s antitumor effects in RCC models and provide a rationale for further evaluation in physiologically relevant *in vivo* systems and with formal pharmacokinetic and toxicity studies.

## 1 Introduction

Despite significant advancements in molecular profiling and targeted therapies for renal cell carcinoma (RCC) in the past decade, this malignancy persists as one of the most therapy-resistant cancers and a leading contributor to global cancer mortality (Miller et al., 2022). Recent studies reveal that approximately 30% of RCC patients develop resistance to first-line tyrosine kinase inhibitors like sunitinib within 12 months of treatment initiation (Jonasch et al., 2021). This acquired resistance, mediated through complex mechanisms involving epigenetic modifications and tumor microenvironment remodeling, underscores the urgent need for novel therapeutic strategies. Contemporary drug development initiatives for RCC have prioritized the development of therapeutic agents demonstrating three critical characteristics: potent antitumor activity, favorable safety profiles, and economic viability. Emerging evidence from phase II/III clinical trials reveals that 41% of RCC patients currently integrate traditional Chinese medicine (TCM) formulations into their therapeutic regimens as complementary interventions (Chen et al., 2022). Mechanistic studies indicate that TCM-derived bioactive compounds exert multi-target effects through tumor microenvironment modulation and angiogenesis inhibition (Xu et al., 2020), potentially synergizing with VEGF-targeted therapies and immune checkpoint inhibitors (Li et al., 2022). Traditional Chinese medicine may effectively manage or reduce the toxic side effects of targeted therapies and bioimmunotherapy, thereby improving the quality of life and extending the survival of patients with intermediate to advanced RCC. Therefore, introducing effective adjuvant therapies for postoperative management and recurrence prevention is crucial to enhancing the current treatment strategies for RCC.

Among these, Compound Kushen Injection (CKI) and Kang’ai Injection (KAI) are two commonly employed adjuvant therapies for advanced RCC in clinical practice. Previous studies have indicated that the combination of CKI with sorafenib and interleukin-2 not only improves the quality of life and immune function of patients but also offers substantial clinical value in the treatment of advanced RCC (Xiao-bei et al., 2015). The combination of KAI with concurrent radiotherapy and chemotherapy can significantly improve short-term efficacy in patients with advanced cervical cancer, while also reducing the incidence of chemotherapy-related side effects (Hongmei et al., 2020). However, there are currently no studies elucidating the specific mechanisms by which these two herbal injections exert their effects in the treatment of RCC. We treated renal cancer cells with CKI and KAI separately and conducted gene sequencing, which identified the TOR1AIP1 gene as having the most significant alterations. Although TOR1AIP1 has been implicated by bioinformatics analyses in several malignancies and appears in pan-cancer resources, experimental validation and mechanistic dissection in RCC remain sparse. Our study addresses this gap by providing *in vitro*/*in vivo* functional evidence and a mechanistic link to JNK signaling, as well as pharmacologic modulation via oxymatrine. We further screened two herbal-derived bioactive compounds, and identified that oxymatrine (OMT) demonstrated dual functionality by significantly inhibiting renal cancer cell proliferation while upregulating TOR1AIP1 expression, which led to its selection for subsequent mechanistic investigations.

An increasing body of research highlights the diverse pharmacological activities of OMT, with its anticancer efficacy being associated with its ability to inhibit cell proliferation, invasion, and epithelial-mesenchymal transition (Chen et al., 2019; Guo et al., 2015). OMT exhibits antitumor potential in a variety of cancers by engaging multiple signaling pathways, including the inhibition of proliferation, induction of apoptosis, suppression of angiogenesis, prevention of metastasis, and enhancement of sensitivity to chemotherapeutic agents, thereby attracting considerable interest (Guo et al., 2015; Liu et al., 2016; Wu et al., 2017). Several studies have demonstrated that the proliferation of various malignant tumor cells, including those of lung, breast, and colon cancers, is affected by OMT and shows a negative correlation with its concentration (Zhou et al., 2019; Liang et al., 2023). Relevant studies have indicated that OMT(8 mg/mL) inhibits the migration and invasion of RCC cells by targeting β-linker proteins (Jin et al., 2020), In our subsequent experiments, we will reference this concentration of OMT. Consistent with prior RCC studies utilizing 8 mg/mL OMT to suppress malignant behaviors, we employed 8 mg/mL OMT to dissect TOR1AIP1–JNK signaling *in vitro*. However, the underlying mechanisms of OMT’s antitumor activity in RCC development remain largely unexplored.

Torsin 1A interacting protein 1 (TOR1AIP1), also referred to as LAP1, is a single-channel membrane protein located in the inner nuclear membrane, playing a crucial role in maintaining the integrity of the nuclear membrane. First identified in 1988 in rat liver samples, it was initially considered an integral protein of the inner nuclear membrane, associated with nuclear fibrillar proteins (Senior and Gerace, 1988). Erceylan et al. concluded that TOR1AIP1 serves as a potential prognostic marker for ovarian and breast cancer, based on combined expression and survival analyses (Erceylan Ö, Savaş and Göv, 2021). However, the aforementioned studies exploring the correlation between TOR1AIP1 and tumor development are limited to bioinformatics predictive analysis, lacking both histological validation and molecular biology insights. Furthermore, the role and mechanism of this gene in the development of RCC remain uninvestigated.

Our investigation reveals novel mechanisms underlying OMT-mediated suppression of RCC progression. Building upon established evidence of OMT’s anti-proliferative and anti-metastatic properties, we demonstrate that TOR1AIP1 serves as the pivotal mediator of these therapeutic effects. Crucially, genetic overexpression of TOR1AIP1 recapitulated OMT’s pharmacological actions by suppressing malignant phenotypes in Caki-1 and 786-O cell lines through JNK signaling pathway inactivation. This functional convergence was evidenced by coordinated inhibition of proliferation, migration, and invasion capacity following TOR1AIP1 upregulation.

## 2 Materials and methods

### 2.1 Data collection and analysis

Tissue samples were obtained from 100 patients diagnosed with RCC between January 2016 and January 2021 at the Affiliated Hospital of Qingdao University. The study adhered to the guiding principles of the Declaration of Helsinki and received approval from the Ethics Committee of the Affiliated Hospital of Qingdao University (Approval No. QYFYWILL26556). The inclusion criteria were as follows: renal cancer confirmed through clinical evaluation, imaging, and pathological analysis. Patients not diagnosed via standard procedures or with a history of other malignancies were excluded from the study. Cancerous tissue was collected from the tumor site, while normal kidney tissue, used as a control, was obtained from an area 5 cm surrounding the tumor lesion. Portions of the fresh tissue specimens were lysed for protein extraction, and TOR1AIP1 expression was analyzed using Western blotting. The remaining tissue was preserved by freezing in liquid nitrogen.

### 2.2 Cell lines and cell culture

Human renal cancer cell lines (Caki-1, 786-O, OS-RC-2, ACHN, 769P, and A498) and the human proximal tubular epithelial cell line HK2 were obtained from the Laboratory of Qingdao University Hospital. These cell lines were cultured in RPMI-1640 and DMEM media, respectively, supplemented with 10% fetal bovine serum, 1% penicillin, and streptomycin. They were maintained in a humidified incubator with 5% CO_2_ at 37 °C and harvested using 0.25% trypsin-EDTA (ethylenediaminetetraacetic acid) (Gibco, China). All cells were stored in liquid nitrogen containers.

### 2.3 Cell transfections, and selection of stable cell lines

Based on the expression levels of TOR1AIP1 in various RCC cell lines, we selected four cell lines—Caki-1, 786-O, OS-RC-2, and A498—to establish stable TOR1AIP1 overexpression or knockdown models. This selection aimed to mitigate the impact of endogenous TOR1AIP1 expression on the effects of recombinant lentiviral transfection. Notably, Caki-1 and 786-O exhibited the lowest TOR1AIP1 expression levels, while OS-RC-2 and A498 showed the highest. Seventy-two hours post-infection, cells were treated with 2 μg/mL puromycin for 7 days to generate stable cell lines. The expression levels of TOR1AIP1 were confirmed via Western blotting. These stable cell lines were subsequently stored in liquid nitrogen containers.

### 2.4 Cell viability assay

RCC cells were seeded into 96-well plates at a density of approximately 1 × 10^3^ cells per well in 100 μL of complete medium supplemented with 10% fetal bovine serum. At specific time points (24 h, 48 h, 72 h, and 96 h), 10 μL of CCK-8 reagent was added to each well and incubated for 2 h, according to the manufacturer’s instructions (Dojindo, Japan). Absorbance was measured at 450 nm using a microplate reader (BioTek Elx9808, United States).

### 2.5 Colony formation assay

To assess the colony-forming potential, transfected cells were seeded in triplicate into 6-well plates at a density of 1,000 cells per well. The cells were incubated at 37 °C for 14 days to facilitate colony formation. After incubation, the colonies were washed twice with phosphate-buffered saline (PBS) and stained with a 0.1% (w/v) crystal violet solution (Beyotime Biotechnology, China). Colonies consisting of at least 50 cells were counted using a microscope. Each experiment was performed in triplicate to ensure the reproducibility and reliability of the results.

### 2.6 Wound healing assay

RCC cells from each experimental group were seeded into 6-well plates and cultured for 48–72 h. Once the cells reached approximately 80%–90% confluency, a sterile 20-μL pipette tip was used to create a linear scratch in the monolayer to simulate a wound. The culture medium was then removed, and the cells were washed 2-3 times with phosphate-buffered saline (PBS) to clear away any debris. The cells were subsequently incubated in a serum-free medium. Cell migration was tracked by capturing images at 0, 6, and 12 h post-scratch using an inverted microscope. The migration area (μm^2^) was quantitatively analyzed with ImageJ software.

### 2.7 Transwell migration assay

RCC cells from each experimental group were harvested and counted, with 1 × 10^4^ cells seeded in a serum-free medium onto an 8 μm pore-size membrane in the upper compartment of a Boyden chamber (Corning Costar, NY, United States) within a 24-well plate. The lower chamber was filled with complete medium containing 10% fetal bovine serum. After a 24-h incubation period, non-migratory cells on the upper side of the membrane were carefully removed. Cells that had migrated to the lower surface of the membrane were fixed with 4% paraformaldehyde and stained with crystal violet solution (Sigma, United States). The number of cells that traversed the membrane was counted using an inverted microscope (Olympus, Japan) in three randomly selected fields per chamber.

### 2.8 Transwell invasion assay

RCC cells from each experimental group (2 × 10^4^ cells per chamber) were seeded into the upper compartments of Boyden chambers, which were pre-coated with 100 μL of 10% matrix gel, in a serum-free medium. The lower compartments were filled with complete medium containing 10% fetal bovine serum. After a 24-h incubation period, cells remaining on the upper side of each membrane were carefully removed. Cells that had migrated to the lower side of the membrane were fixed and stained, following the procedure used in a migration assay. The number of infiltrating cells was counted in three randomly selected fields of view using an inverted microscope (Olympus, Japan).

### 2.9 Western blot analysis

Total protein was extracted using RIPA lysis buffer supplemented with protease and phosphatase inhibitor cocktails. Protein concentration was determined using a BCA protein assay kit. Subsequently, 25 µg of protein from each sample was subjected to 10% sodium dodecyl sulfate-polyacrylamide gel electrophoresis (SDS-PAGE). Post-electrophoresis, proteins were transferred onto PVDF membranes, which were then blocked with 5% non-fat dry milk. The membranes were incubated with primary antibodies against TOR1AIP1, phosphorylated p38, p38, phosphorylated ERK 1/2, ERK 1/2, phosphorylated JNK, JNK, and GAPDH at 4 °C for 12 h. Following this, the membranes were incubated with horseradish peroxidase (HRP)-conjugated secondary antibodies for 1 h at room temperature. Protein bands were visualized using an ECL chemiluminescent substrate, and images were captured with an imaging system. Densitometric analysis of the bands was conducted using ImageJ software.

### 2.10 Xenografted tumor models

BALB/c nude mice (6–8 weeks old) were procured from Jinan Pengyue Laboratory Animal Center (China) and randomly assigned into various experimental groups, with 5 mice per group. Each mouse was subcutaneously injected with 2 × 10^7^ cells from the following lines: Caki-1/Vector, Caki-1/TOR1AIP1, 786-O/Vector, 786-O/TOR1AIP1, OS-RC-2/shRNA-Vector, OS-RC-2/TOR1AIP1-sh#1, OS-RC-2/TOR1AIP1-sh#2, A498/shRNA-Vector, A498/TOR1AIP1-sh#1, and A498/TOR1AIP1-sh#2. The animals were maintained in a specific pathogen-free (SPF) facility for 40 days. To study the role of TOR1AIP1 in the *in vivo* OMT treatment of RCC, 2 × 10^7^ cells transfected with either shRNA vectors or TOR1AIP1-sh#1 lentivirus (OS-RC-2 and A498) were injected subcutaneously into the right flanks of BALB/c nude mice. The mice were divided into four groups (n = 5 per group): (1) shRNA-vector + DMSO; (2) TOR1AIP1-sh#1 + DMSO; (3) shRNA-vector + OMT; and (4) TOR1AIP1-sh#1 + OMT. When tumors in the shRNA-vector + DMSO group reached a diameter of 5 mm, either OMT (20 mg/kg) or DMSO was administered via peritumoral subcutaneous injections twice weekly for 5 weeks. Tumor volume was calculated using the formula: *V* = (*L* × *W* ^2^)/2, where *L* is the longest axis and *W* the shortest. Upon conclusion of the study, all mice were euthanized, and the tumors were excised for histological analysis. OMT was administered via peritumoral subcutaneous injections to establish proof-of-mechanism. Subsequent studies will evaluate systemic administration to enhance translational relevance and enable PK/tox assessments. All experimental procedures involving animals were reviewed and approved by the Animal Research Ethics Committee of the Affiliated Hospital of Qingdao University (AHQU-MAL20230818yxc).

### 2.11 Statistical analysis

For bioinformatic analysis, statistical analyses were conducted using the R programming language (version 3.6.3). For bioassay validation, comparisons between two groups were assessed with a two-tailed Student’s t-test using GraphPad Prism 7.0 (GraphPad, San Diego, CA, United States). A *p*-value less than 0.05 was considered indicative of statistical significance.

## 3 Results

### 3.1 OMT treatment regulating TOR1AIP1 level, inhibits cell viability, migration, and invasion in renal carcinoma cells

**FIGURE 1:**
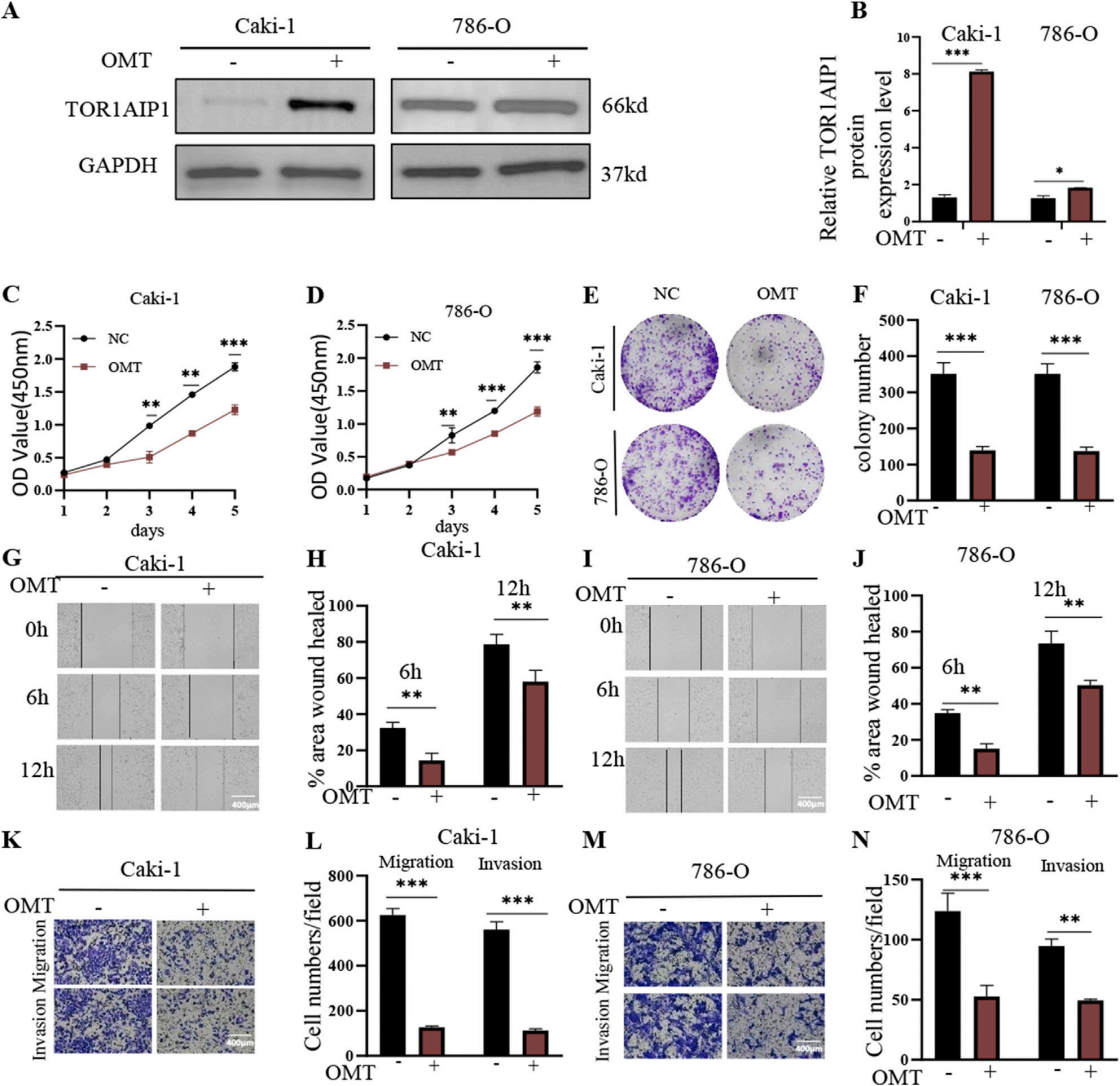
OMT treatment upregulates TOR1AIP1 expression, induces phenotypic changes in Caki-1 and 786-O cells, **(A)** Western blot analysis of TOR1AIP1 expression level in Caki-1 and 786-O cells with or without OMT treatment. **(B)** Representative blots showing upregulation of TOR1AIP1 protein expression in OMT-treated groups. **(C,D)** The viability of Caki-1 and 786-O cells was measured at the indicated times by the CCK-8 assay. **(E,F)** Effect of OMT on colony formation in Caki-1 and 786-O cells. **(G–J)** Wound healing assay for Caki-1 and 786-O cells. **(G,I)** Representative images showing wound closure at 0, 6, and 12 h with and without OMT treatment. **(H,J)** Quantitative analysis of wound closure percentage indicating significantly reduced migration in OMT-treated groups. **(K–N)** Transwell assays of Caki-1 and 786-O cells. **(K,M)** Quantitative results of migration and invasion assays under OMT treatment. **(L,N)** Representative microscopy images showing suppression of cell migration and invasion by OMT. By comparison with vehicle control group, *P < 0.05, **P < 0.01 and ***P < 0.001. Data (means ± SEM) were representative of three separate experiments with similar results (n = 3 independent experiments).

Western blot analysis revealed that treatment with CKI or KAI significantly upregulated TOR1AIP1 protein levels in both Caki-1 and 786-O cells (Supplementary Figure S1). Similarly, OMT monotherapy also increased TOR1AIP1 expression in these renal cell carcinoma models (Figures 1A,B). OMT significantly inhibited the viability of both Caki-1 and 786-O cells in a time-dependent manner as assessed by the CCK-8 assay (Figures 1C,D). In addition, OMT exposure suppressed colony formation ability in these cells, further illustrating its inhibitory effects on cell proliferation (Figures 1E,F). Wound healing assays demonstrated that OMT treatment markedly impaired the migration capacity of both Caki-1 and 786-O cells, as indicated by delayed wound closure over 6 and 12 h (Figures 1G–J). Furthermore, transwell migration and invasion assays revealed that OMT significantly reduced the migratory and invasive potential of Caki-1 and 786-O cells compared to the control group (Figures 1K–N).

### 3.2 TOR1AIP1 expression correlates with clinical outcomes and protein downregulation in tumor tissues

**FIGURE 2:**
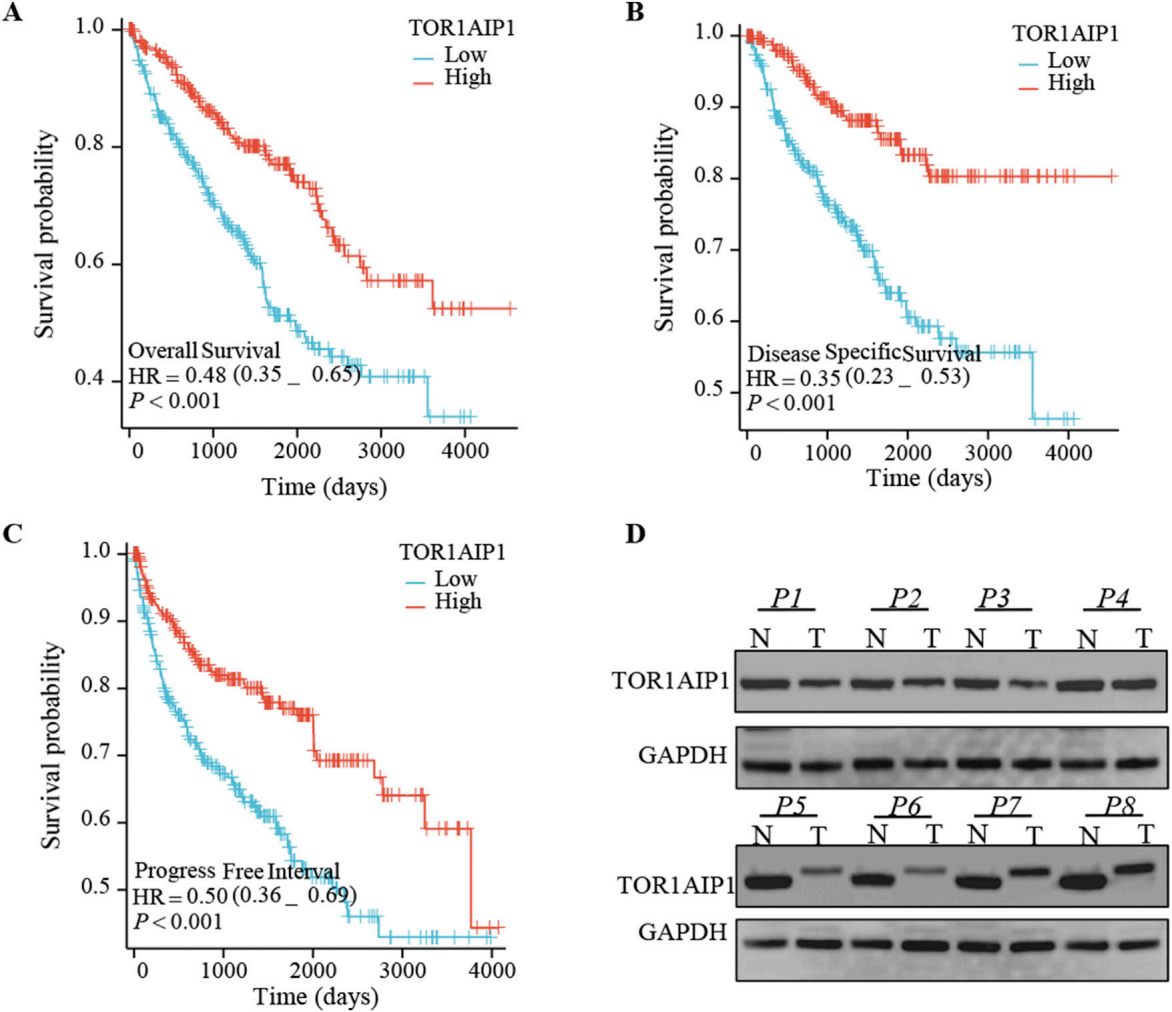
The impact of TOR1AIP1 expression levels on patient survival and protein expression in tumor tissues. **(A–C)** Kaplan-Meier survival curves comparing the survival outcomes of patients with low and high TOR1AIP1 expression in terms of overall survival (OS) **(A)**, disease-specific survival (DSS) **(B)**, and progression-free interval (PFI) **(C)**. Hazard ratios (HR) with 95% confidence intervals (CIs) and p-values are provided. Patients with high TOR1AIP1 expression demonstrated significantly better survival outcomes across all categories (*P* < 0.001 for OS, DSS, and PFI).**(D)** Western blot analysis of eight paired clinical specimens (P1-P8) revealed tumor-specific downregulation of TOR1AIP1 protein expression, with GAPDH serving as the loading control. Comparative analysis demonstrated consistently reduced TOR1AIP1 levels in tumor tissues relative to matched normal counterparts.

Kaplan-Meier survival analyses revealed that higher TOR1AIP1 expression levels were significantly associated with improved survival outcomes in cancer patients. Specifically, patients with high TOR1AIP1 expression exhibited better overall survival (OS) (HR = 0.48, 95% CI: 0.35–0.65, *P* < 0.001) (Figure 2A), disease-specific survival (DSS) (HR = 0.35, 95% CI: 0.23–0.53, *P* < 0.001) (Figure 2B), and progression-free interval (PFI) (HR = 0.50, 95% CI: 0.36–0.69, *P* < 0.001) (Figure 2C). These findings highlight a robust correlation between high TOR1AIP1 expression and favorable clinical outcomes. Furthermore, Western blot analysis of eight paired tumor and normal clinical samples demonstrated significant downregulation of TOR1AIP1 protein expression in tumor tissues compared to their normal counterparts, with GAPDH serving as a loading control (Figure 2D). Collectively, these results indicate that loss of TOR1AIP1 expression in tumors might contribute to disease progression, while higher expression levels are predictive of better prognoses.

### 3.3 TOR1AIP1 suppresses RCC progression *in vitro* and *in vivo*

**FIGURE 3:**
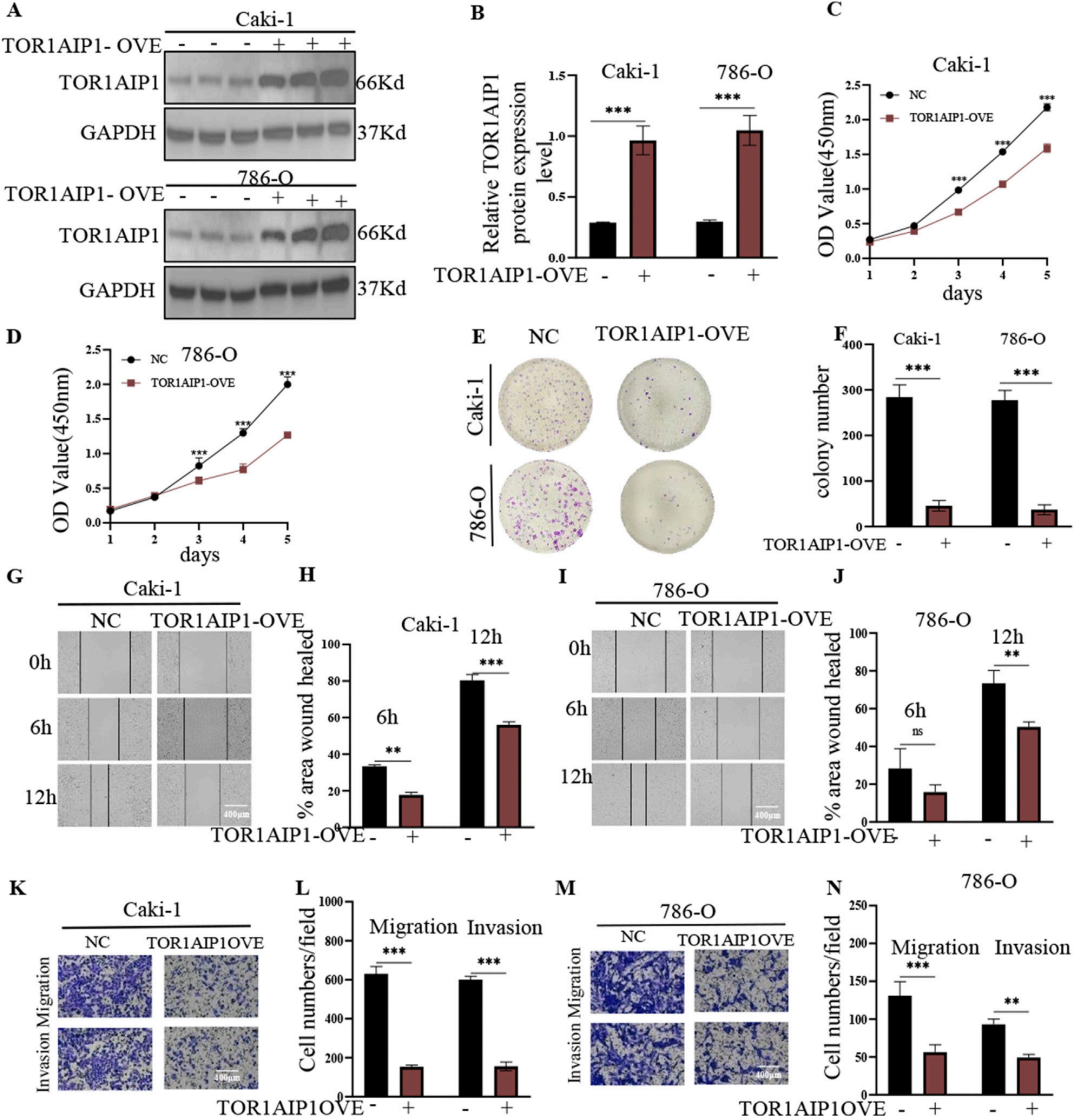
TOR1AIP1 over-expression suppresses proliferation, migration and invasion of RCC cells *in vitro*. (**A,B)** The interference efficiency of lentivirus carrying TOR1AIP1 was verified by Western blot. **(C,D)** The viability of Caki-1 and 786-O cells was measured at the indicated times by the CCK-8 assay. **(E,F)** Effect of TOR1AIP1 overexpression on colony formation in renal cancer cells. **(G–J)** Effect of TOR1AIP1 overexpression on the migration ability of renal cancer cells. **(K–N)** Metastatic and invasive properties of TOR1AIP1 overexpressing were determined by transwell assay.By comparison with vehicle control group, *P < 0.05, **P < 0.01 and ***P < 0.001. Data (means ± SEM) were representative of three separate experiments with similar results (n = 3 independent experiments).

**FIGURE 4:**
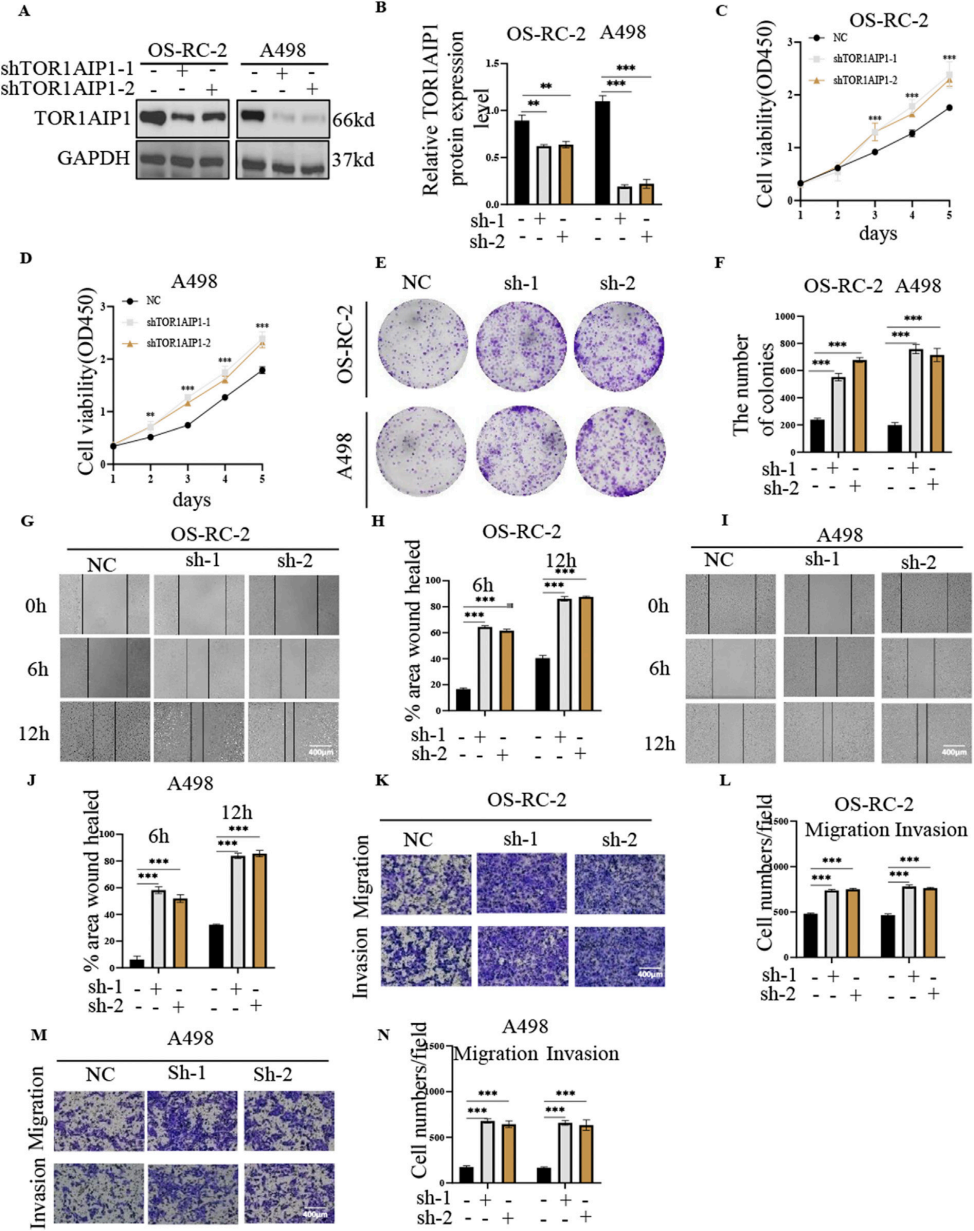
TOR1AIP1 knockdown promotes RCC proliferation, invasion *in vitro*. **(A,B)** Validation of knockdown efficiency of targeted TOR1AIP1 in OS-RC-2 and A498 cells by Western blot. **(C,D)** The viability of OS-RC-2 and A498 cells was measured at the indicated times by the CCK-8 assay. **(E,F)** Effect of TOR1AIP1 knockdown on colony formation in renal cancer cells. **(G–J)** Effect of TOR1AIP1 knockdown on the migration ability of renal cancer cells. **(K–N)** Metastatic and invasive properties of TOR1AIP1 knockdown OS-RC-2 and A498 cells were determined by transwell assay. By comparison with vehicle control group, *P < 0.05, **P < 0.01 and ***P < 0.001. Data (means ± SEM) were representative of three separate experiments with similar results (n = 3 independent experiments).

**FIGURE 5:**
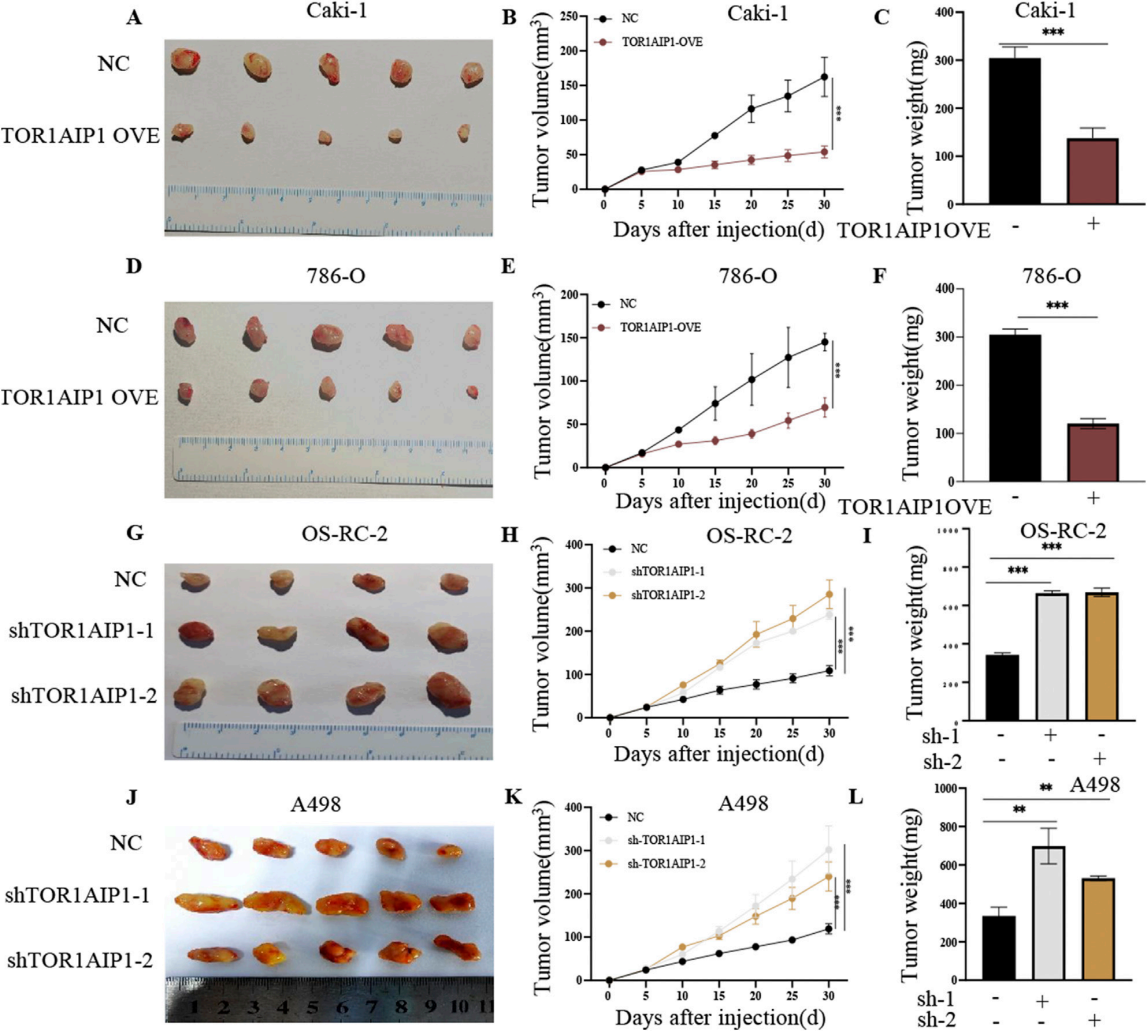
TOR1AIP1 regulates renal cancer progression in a xenograft mouse model. **(A–F)** The *in vivo* effect of TOR1AIP1 overexpression on renal cancer progression. **(A,D)** Gross images of subcutaneous tumors derived from the xenograft model using Caki-1 and 786-O cells with TOR1AIP1 overexpression and negative controls (NC). **(B,E)** Tumor growth curves of different groups. Tumor volume (mm^3^) was measured every 5 days following tumor cell inoculation. **(C,F)** Tumor weights measured at the endpoint of the experiments. **(G–L)** The *in vivo* effect of TOR1AIP1 knockdown on renal cancer progression. **(G,J)** Gross images of subcutaneous tumors derived from the xenograft model using OS-RC-2 and A498 cells with TOR1AIP1 knockdown (shTOR1AIP1-1, shTOR1AIP1-2) and negative controls (NC). **(H,K)** Tumor growth curves of different groups. Tumor volume (mm^3^) was measured every 5 days following tumor cell inoculation. **(I,L)** Tumor weights measured at the endpoint of the experiments. Data are presented as means SEM. unpaired Student’s t-test. *p < 0.05 and ***p < 0.001.

Western blot analysis revealed differential TOR1AIP1 protein expression across renal cancer cell lines, with Caki-1 and 786-O exhibiting the lowest baseline levels compared to OS-RC-2 and A498 cells (Supplementary Figure S2). Functional characterization through gain- and loss-of-function approaches demonstrated TOR1AIP1’s tumor-suppressive role. Lentivirus-mediated overexpression in Caki-1 and 786-O cells (Figures 3A,B) significantly attenuated malignant phenotypes: CCK-8 assays showed reduced cell viability (Figures 3C,D), while colony formation, wound healing, and transwell assays revealed impaired proliferative, migratory, and invasive capacities (Figures 3E–N). Conversely, shRNA-mediated knockdown in OS-RC-2 and A498 cells (Figures 4A,B) enhanced tumorigenic potential, with accelerated proliferation (Figures 4C–F), improved wound closure (Figures 4G–J), and increased migration/invasion (Figures 4K–N). *In vivo* validation using subcutaneous xenografts confirmed these findings. TOR1AIP1-overexpressing Caki-1 and 786-O cells generated significantly smaller tumors than controls, with delayed growth kinetics and reduced terminal tumor weights (Figures 5A–F). Reciprocally, TOR1AIP1-silenced OS-RC-2 and A498 cells produced larger tumors displaying accelerated growth rates and increased mass (Figures 5G–L). These complementary experimental approaches establish TOR1AIP1 as a critical suppressor of RCC progression through coordinated regulation of tumor cell proliferation and metastatic potential.

### 3.4 OMT inhibits renal cancer progression by enhancing TOR1AIP1 expression *in vitro* and *in vivo*

**FIGURE 6:**
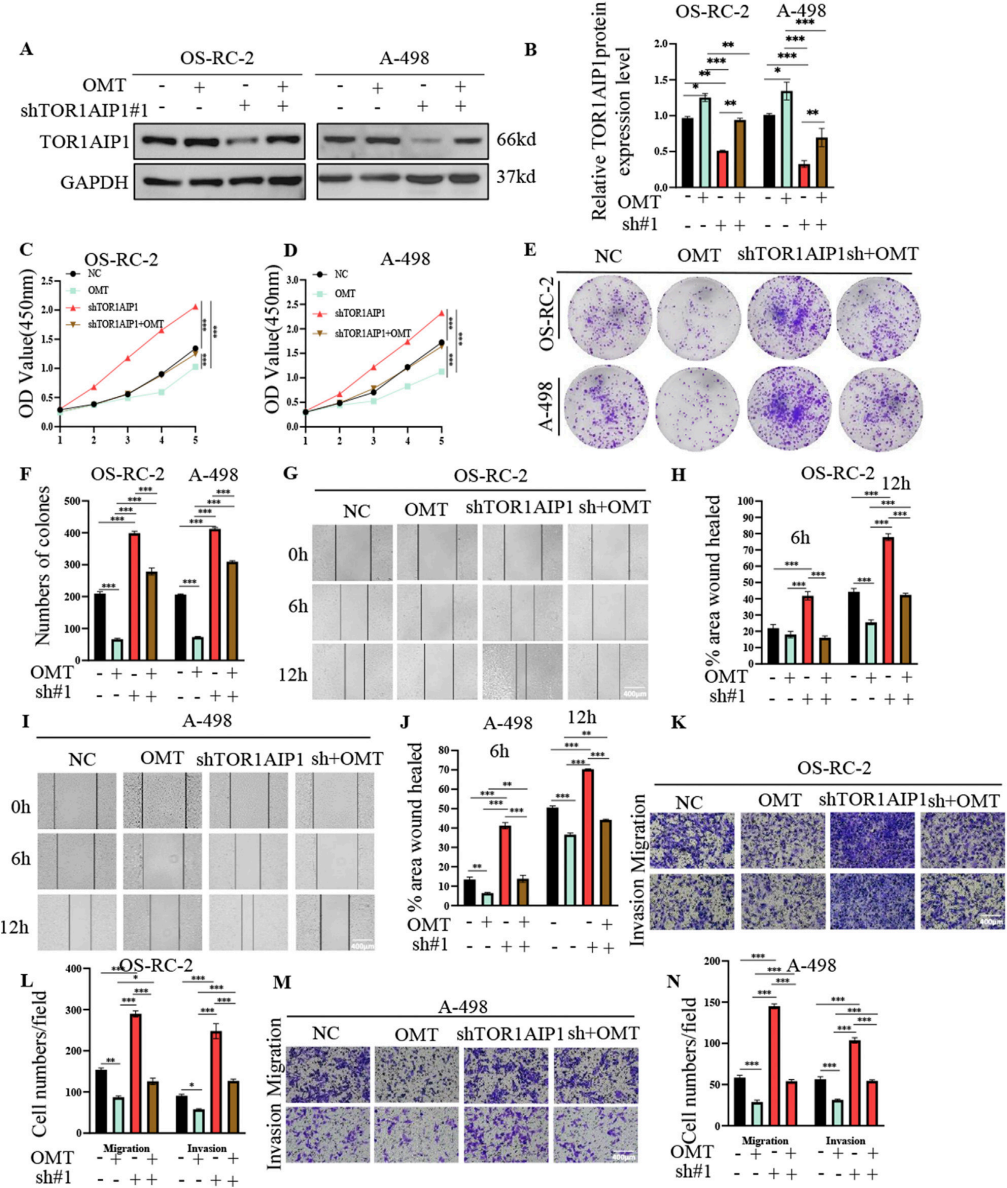
OMT Suppresses Renal Cancer Progression by Promoting TOR1AIP1 Expression. OSRC-2 and A498 cells after TOR1AIP1 knockdown were treated with OMT for 48 h **(A,B)** TOR1AIP1 protein levels were determined by Western blot in OS-RC-2 and A498 cells. **(C,D)** Cell viability was measured by CCK-8 assay. **(E,F)** Plate cloning assays displaying the number of cell colonies in each treatment group. Representative images and quantification are shown. **(G,I)** Wound-healing assays were performed to evaluate cell migration ability in OS-RC-2 and A498 cells after OMT treatment with or without TOR1AIP1 knockdown. Images were taken at 0 h, 6 h, and 12 h, and the wound-healed area percentages were calculated. **(H,J)** Quantification of wound-healing results for each group. **(K,M)** Transwell invasion assays were used to evaluate the effects of OMT, with or without TOR1AIP1 knockdown, on cell invasive ability in OS-RC-2 and A498 cells. Representative images and quantification are shown. **(L,N)** Quantification of invasive cells per field under the different treatment conditions. By comparison with vehicle control group, *P < 0.05, **P < 0.01 and ***P < 0.001. Data (means ± SEM) were representative of three separate experiments with similar results (n = 3 independent experiments).

**FIGURE 7:**
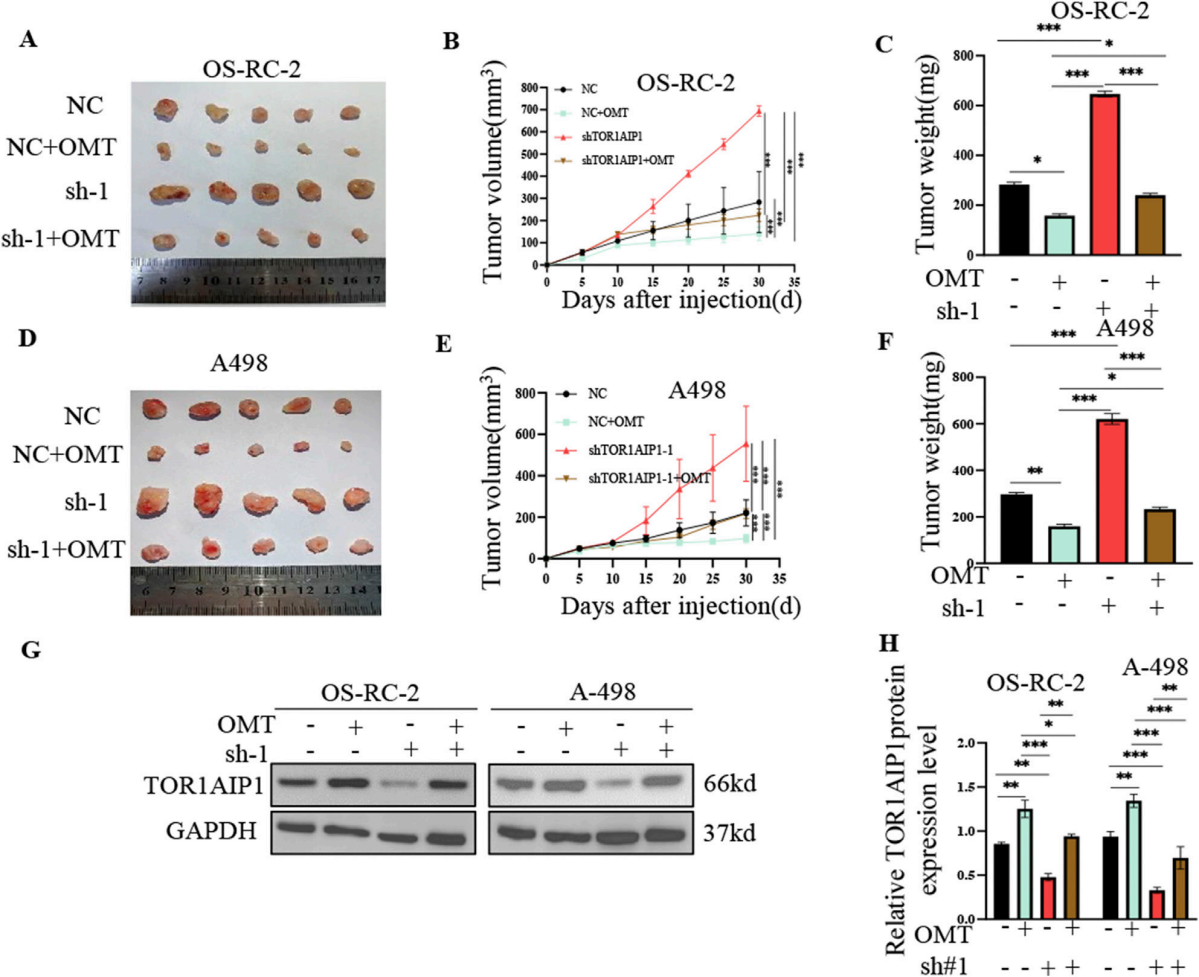
OMT Suppresses Renal Cancer Progression by Promoting TOR1AIP1 Expression *in vivo*. **(A,D)** The xenografts of OSRC-2 and A498 cells were harvested at 30 days after transplantation, and the morphology was photographed. **(B,E)** Tumor diameters were measured at a regular interval of 5 days for up to 30 days and the tumor volume was calculated. **(C,F)** Tumor weight of each group was measured at the end of the experiments. **(G)** The protein expression of TOR1AIP1 in tumor tissues determined by Western blot. **(H)** Relative TOR1AIP1 protein level in G was calculated. Data were generated in subcutaneous xenografts; translational inference to angiogenesis- or metastasis-dependent contexts requires orthotopic and metastasis models. By comparison with vehicle control group, *P < 0.05, **P < 0.01 and ***P < 0.001. Data (means ± SEM) were representative of three separate experiments with similar results (n = 3 independent experiments).

Oxymatrine (OMT, 8 mg/mL, 48 h) treatment in OS-RC-2 and A498 renal cancer cells markedly upregulated TOR1AIP1 protein expression (Figures 6A,B). Functional rescue experiments using TOR1AIP1-knockdown models demonstrated OMT-mediated tumor suppression predominantly through TOR1AIP1 regulation. Both CCK-8 viability assays and colony formation analyses revealed that OMT significantly attenuated cancer cell proliferation, an effect substantially reversed by TOR1AIP1 silencing (Figures 6C–F). Concordantly, OMT suppressed wound closure rates (Figures 6G–J) and transwell invasiveness (Figures 6K–N) in control cells, whereas TOR1AIP1 knockdown partially restored migratory and invasive capacities. These findings were recapitulated in xenograft models (n = 5/group) using TOR1AIP1-modified OS-RC-2 and A498 cells. Systemic OMT administration significantly reduced tumor volume progression and end-stage tumor mass in control xenografts (NC + OMT vs NC, Figures 7A–F). Notably, this antitumor efficacy was markedly diminished in TOR1AIP1-silenced tumors (sh-TOR1AIP1 + OMT vs sh-TOR1AIP1). Western blot validation confirmed OMT-induced TOR1AIP1 upregulation in tumor lysates, with suppressed baseline expression in knockdown groups (Figures 7G,H). These complementary data establish TOR1AIP1 as the critical molecular mediator of OMT’s anticancer activity in renal carcinoma models. We note that these *in vivo* findings were obtained in subcutaneous xenograft models that do not fully recapitulate the angiogenesis dependence and metastatic microenvironments characteristic of human RCC.

### 3.5 TOR1AIP1 inhibits renal cancer progression by regulating the JNK signaling pathway, *in vitro* and *in vivo*

**FIGURE 8:**
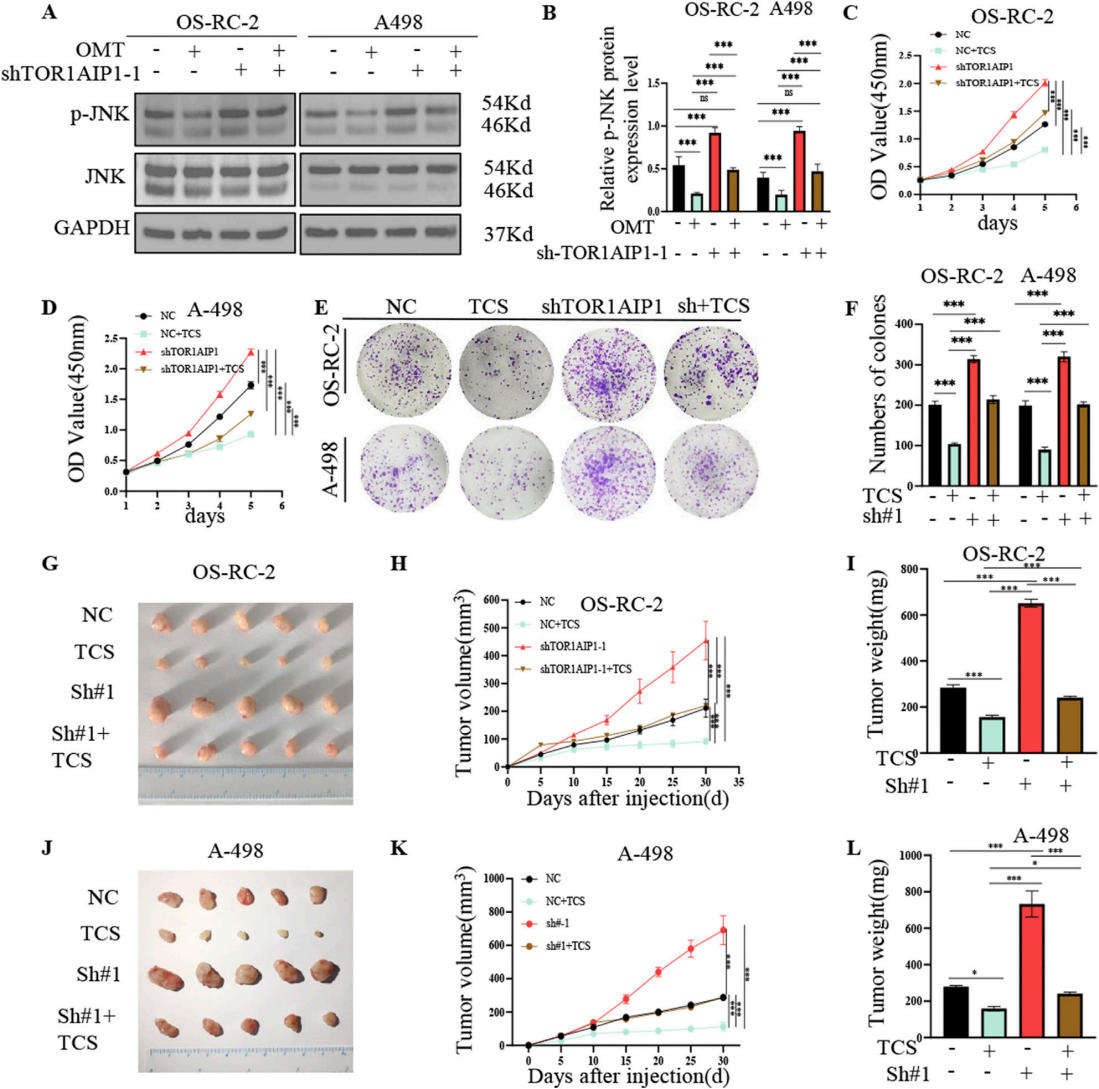
Inhibitor of JNK signaling pathway reverses kidney cancer progression due to TOR1AIP1 knockdown *in vitro* and *vivo*. **(A,B)** Western blot analysis showing the effect of OMT on JNK signaling in OS-RC-2 and A498 cells. OMT treatment reduces p-JNK protein levels, indicating pathway inhibition, while knockdown of TOR1AIP1 reverses this inhibitory effect. **(C–F)** Cell viability and colony formation assays for OS-RC-2 and A498 cells. Upon TOR1AIP1 knockdown, cell proliferation is enhanced. However, treatment with the JNK inhibitor reverses the increased proliferation caused by TOR1AIP1 knockdown in both cell lines, as shown by CCK-8 assay and plate colony-forming assay. **(G-L)** Nude mouse xenograft tumor inhibition experiments. OS-RC-2 and A498 cells with or without TOR1AIP1 knockdown were transplanted into nude mice. Tumor-bearing mice were treated with TCS via peri-tumoral injection, as indicated. Tumor volumes were measured every 5 days, and tumors were excised and weighed at the end of the experiment. TCS injection significantly inhibited tumor growth driven by TOR1AIP1 knockdown compared to untreated groups. Data were generated in subcutaneous xenografts; translational inference to angiogenesis- or metastasis-dependent contexts requires orthotopic and metastasis models. By comparison with vehicle control group, *P < 0.05, ***P < 0.001. Data (means ± SEM) were representative of three separate experiments with similar results (n = 3 independent experiments). Expression of the TOR1AIP1 protein was upregulated in renal cancer cells following treatment with CKI and KAI. **(A–D)** Renal cancer cells Caki-1 and 786-O were treated with CKI and KAI for 48 h, and the TOR1AIP1 protein was upregulated. *P < 0.05 and ***P < 0.001.

OMT treatment significantly inhibited the activation of JNK signaling in OS-RC-2 and A498 cells, as shown by reduced levels of phosphorylated JNK (p-JNK), without affecting p-ERK or p-P38 levels. OMT treatment reduced p-JNK, and in most conditions had limited effects on p-ERK and p-p38,suggesting selective but not exclusive MAPK modulation (Supplementary Figure S3A–F). In contrast, knockdown of TOR1AIP1 using shRNAs led to a significant increase in p-JNK expression, suggesting a regulatory role of TOR1AIP1 in activating this pathway (Supplementary Figure S3G,H). Pharmacological inhibition studies confirmed OMT-mediated suppression of renal cancer progression through JNK signaling modulation. Western blot analysis demonstrated that OMT treatment effectively reversed TOR1AIP1 knockdown-induced p-JNK elevation (Figures 8A,B), prompting subsequent rescue experiments with the specific JNK inhibitor TCS. This mechanistic approach substantiated that JNK pathway activation mediates the oncogenic effects of TOR1AIP1 deficiency. Functional assays, including CCK-8 and colony formation assays, demonstrated that the increased cell proliferation caused by TOR1AIP1 knockdown was significantly suppressed by JNK inhibition (Figures 8C–F). Wound healing and Transwell migration assays were performed,TOR1AIP1 knockdown significantly enhanced the migration and invasion of OS-RC-2 and A498 cells, while TCS treatment reversed these effects (Supplementary Figure S4A–H). *In vivo*, nude mouse xenograft experiments showed that TCS treatment significantly inhibited tumor growth and proliferation in TOR1AIP1 knockdown groups (Figures 8G–L). These findings provide strong evidence that TOR1AIP1 regulates renal cancer progression by modulating JNK signaling, which in turn influences cell migration and invasion.

## 4 Disscusion

This study identifies oxymatrine (OMT) as a promising new treatment option for renal cell carcinoma (RCC) by demonstrating its ability to modulate TOR1AIP1 expression and selectively inhibit the JNK signaling pathway. Our findings indicate that OMT significantly reduces harmful characteristics of RCC in laboratory models, such as cell proliferation, migration, and invasion, particularly in the Caki-1 and 786-O cell lines, while simultaneously increasing the expression of the tumor-suppressing protein TOR1AIP1. Notably, survival analyses using the Kaplan-Meier method revealed a strong correlation between high levels of TOR1AIP1 and better clinical outcomes, with p-values less than 0.001 for overall survival, disease-specific survival, and progression-free interval, which aligns with growing evidence of its role in suppressing tumors across various cancers. Mechanistically, OMT was found to specifically reduce levels of phosphorylated JNK (p-JNK), while predominantly reducing p-JNK, with context-dependent, modest effects on ERK and p38. These findings align with MAPK family crosstalk in RCC and suggest selective—but not exclusive—MAPK modulation by OMT. To strengthen target prioritization, we will perform phospho-proteomics and/or RNA-seq under OMT treatment and TOR1AIP1 perturbation to comprehensively map MAPK and non-MAPK nodes and confirm JNK pathway predominance. This distinction is crucial, as different MAPK subfamilies have varying roles in cancer development (Xu et al., 2018; Blaj et al., 2017; Mishima, Inoue and Hayashi, 2002). The importance of TOR1AIP1 in mediating the anti-RCC effects of OMT was confirmed through experiments that either increased or decreased its expression. Overexpressing TOR1AIP1 produced effects similar to those of OMT treatment, while knocking it down negated the therapeutic benefits, highlighting this nuclear envelope protein as a key player in OMT’s action. This discovery broadens our understanding of TOR1AIP1’s functions beyond its known roles in maintaining nuclear structure and regulating redox processes. Additionally, the inhibition of the JNK pathway in cells treated with OMT supports recent research in liver cancer models, where OMT influenced interactions between tumors and surrounding tissue through MAPK regulation, suggesting that this mechanism may be common across different types of epithelial cancers. Importantly, our research expands the known therapeutic potential of OMT beyond its previously recognized effects.

The tumor-suppressive effects of OMT observed in renal cell carcinoma not only align with but also significantly broaden its known therapeutic benefits across various cancers. Earlier research on lung, breast, and colon cancers demonstrated OMT’s ability to inhibit tumor growth and spread by influencing apoptosis regulators, such as the Bcl-2/Bax axis, and inflammatory mediators like the NF-κB/IL-6 pathway (Jung et al., 2019; Liu et al., 2019). However, our study introduces TOR1AIP1 as a new molecular mediator and identifies the JNK pathway as the main signaling pathway affected by OMT. This specific mechanism differs from previous findings that reported a more general inhibition of the MAPK pathway by OMT in hepatocellular carcinoma or its modulation of the TGF-β/Smad3 axis in pancreatic cancer models (Suwanabol et al., 2012; Xu et al., 2013; Ooshima et al., 2019). Notably, OMT selectively downregulates p-JNK without affecting ERK or p38 phosphorylation, highlighting a new level of precision in OMT’s molecular targeting. This specificity may account for its lower cytotoxicity in non-cancerous cells compared to traditional kinase inhibitors.

Our findings significantly enhance the biological understanding of TOR1AIP1, a protein located at the nuclear envelope, which has previously been linked to survival analyses in ovarian and breast cancers (Cossins et al., 2020; Lessel et al., 2020). Our study reveals TOR1AIP1 active role in the pathogenesis of RCC through both gain- and loss-of-function experiments. We observed that the anti-tumor effects of OMT were replicated through the overexpression of TOR1AIP1, and importantly, these therapeutic benefits were completely reversed when TOR1AIP1 was knocked down. This positions TOR1AIP1 as a crucial executor in the progression of RCC rather than merely a bystander. By elevating TOR1AIP1 from a biomarker to a functional mediator, we address longstanding questions regarding its role in malignant transformation and broaden its potential for therapeutic targeting beyond cancers where TOR1AIP1 has established survival correlations.

The identification of JNK pathway inhibition as the primary mechanism in RCC stands in contrast to the effects of OMT in other cancers, where it often influences overlapping pathways through mechanisms dependent on reactive oxygen species (ROS) or interactions within the immune microenvironment (Hu et al., 2025; Jung et al., 2019; Halim et al., 2019). This specificity in pathway selection may be attributed to the unique metabolic requirements of RCC or the distinct activation patterns of MAPK subfamilies in renal cancers compared to those in liver or lung cancers (Hegazy and Emam, 2015; Salehiniya et al., 2022). Notably, our findings indicate that TOR1AIP1 functions as both a sensor and a regulator of JNK signaling fidelity in RCC, establishing a self-reinforcing loop that suppresses tumor growth when activated pharmacologically by OMT. This dual regulatory role—enhancing an endogenous tumor suppressor protein while simultaneously inhibiting a key oncogenic kinase cascade—offers a therapeutic approach that differs from the single-pathway inhibitors currently being explored in clinical trials.

From a translational perspective, these data provide a mechanistic basis to justify further preclinical development, including evaluation in orthotopic and metastasis-competent RCC models and comprehensive pharmacokinetic and toxicity studies, prior to any clinical consideration. OMT may be a candidate for hypothesis-generating combination studies with VEGF-targeted agents or immunotherapy in preclinical models that capture angiogenesis and immune contexture. We will assess OMT combinations with sunitinib and nivolumab in orthotopic and syngeneic/immune-competent models, measuring synergy (Bliss/Loewe), vascular metrics, and TME composition.

This work contributes to a new approach where traditional medicine derivatives are carefully assessed using modern molecular oncology methods. By integrating the diverse anti-cancer effects of OMT into a cohesive model centered around the TOR1AIP1-JNK axis, we create a framework for repurposing other TCM compounds that have unclear mechanisms of action. Given that clinical surveys show a significant number of patients are turning to herbal supplements in the management of RCC, it is crucial to thoroughly investigate their molecular targets. This is essential to avoid negative interactions and to enhance the effectiveness of treatments. Our research not only supports OMT as a well-founded therapeutic option but also identifies TOR1AIP1 as a promising biomarker for categorizing patients in upcoming clinical trials involving JNK pathway inhibitors.

## 5 Limitations and translational considerations

Our *in vivo* data were obtained from subcutaneous xenografts, which do not completely replicate the complex vascularized microenvironment and metastatic niches that are characteristic of RCC. Therefore, it is crucial to validate the antitumor effects of OMT, which operates through TOR1AIP1 upregulation and modulation of the JNK pathway, in more representative models such as orthotopic kidney tumors, angiogenesis-dependent systems, and models of spontaneous or experimental metastasis, such as lung colonization. Additionally, our current dosing method involved peritumoral subcutaneous administration; future research will focus on more clinically relevant systemic administration routes, including intraperitoneal or intravenous methods. We will also investigate exposure–response relationships in both tumor and plasma, as well as confirm pharmacodynamic effects, such as the suppression of intratumoral p-JNK. These steps are vital for establishing the translational relevance of our findings. A limitation of this work is the absence of direct HK-2 cytotoxicity data; our safety rationale is literature-based and will be complemented by future selectivity and PK/PD studies.

## 6 Pharmacokinetics, toxicity, and dosing strategy

The current study did not conduct formal assessments of pharmacokinetics (PK) and toxicity related to OMT in the context of renal cell carcinoma (RCC). To substantiate translational claims, future research should focus on characterizing the absorption, distribution, metabolism, and excretion (ADME) of OMT. This includes determining the maximum tolerated dose, identifying dose-limiting toxicities, and measuring OMT concentrations in both plasma and tumor tissues. Additionally, we aim to correlate exposure levels with pharmacodynamic outcomes, such as the inhibition of p-JNK and modulation of TOR1AIP1, as well as evaluate the antitumor efficacy. Furthermore, we will explore route optimization for systemic administration that is clinically relevant, to better reflect potential applications in clinical settings.

## 7 Conclusion

In conclusion, this study emphasizes the potential of OMT as a promising new treatment for RCC, showing its effectiveness in slowing down tumor growth in both laboratory and animal models. The anticancer properties of OMT are linked to the increased expression of TOR1AIP1, a protein that acts as a tumor suppressor by inhibiting the JNK signaling pathway. When TOR1AIP1 levels were elevated, there was a notable decrease in RCC cell growth, movement, and invasion; conversely, reducing TOR1AIP1 levels negated the positive effects of OMT. Furthermore, an analysis of clinical data revealed a strong connection between high levels of TOR1AIP1 and better outcomes for patients. These results highlight the TOR1AIP1-JNK pathway as a crucial mechanism through which OMT exerts its tumor-suppressive effects, suggesting that the TOR1AIP1–JNK axis is a tractable mechanism for OMT in RCC models. However, formal pharmacokinetic, toxicity, and advanced *in vivo* model studies are required before any clinical inferences can be drawn. Accordingly, we have tempered clinical language throughout and reframed claims to emphasize mechanistic insight and the need for further preclinical validation. Future research should focus on assessing the effectiveness of OMT in combination with other therapies and investigate the role of TOR1AIP1 as a potential prognostic marker for RCC.

## Data Availability

The datasets presented in this study can be found in online repositories. The names of the repository/repositories and accession number(s) can be found in the article/Supplementary Material.
