# Supplementary material for: Oxymatrine inhibited the progression of renal cell carcinoma by increasing TOR1AIP1 expression
Source: Front Pharmacol. 2025 Oct 2;16:1611069. doi: 10.3389/fphar.2025.1611069 (PMC12528049; doi:10.3389/fphar.2025.1611069)

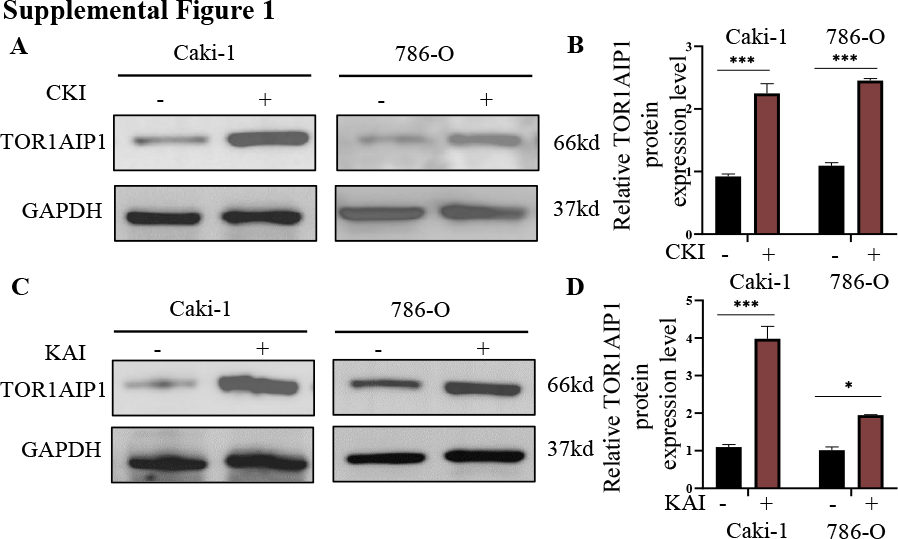


**Expression of the TOR1AIP1 protein was upregulated in renal cancer cells following treatment with CKI and KAI.**

**(A-D)** Renal cancer cells Caki-1 and 786-O were treated with CKI and KAI for 48 hours, and the TOR1AIP1 protein was upregulated.*P < 0.05 and ***P < 0.001.


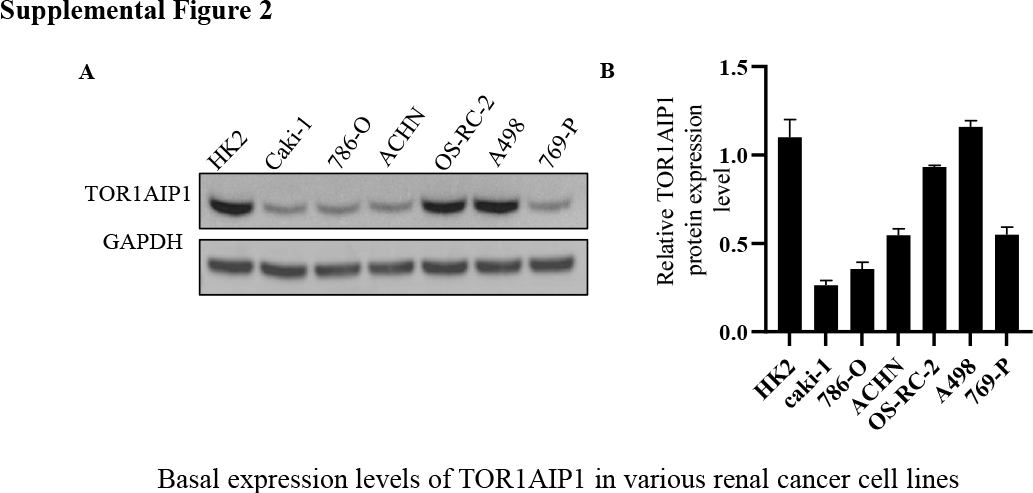


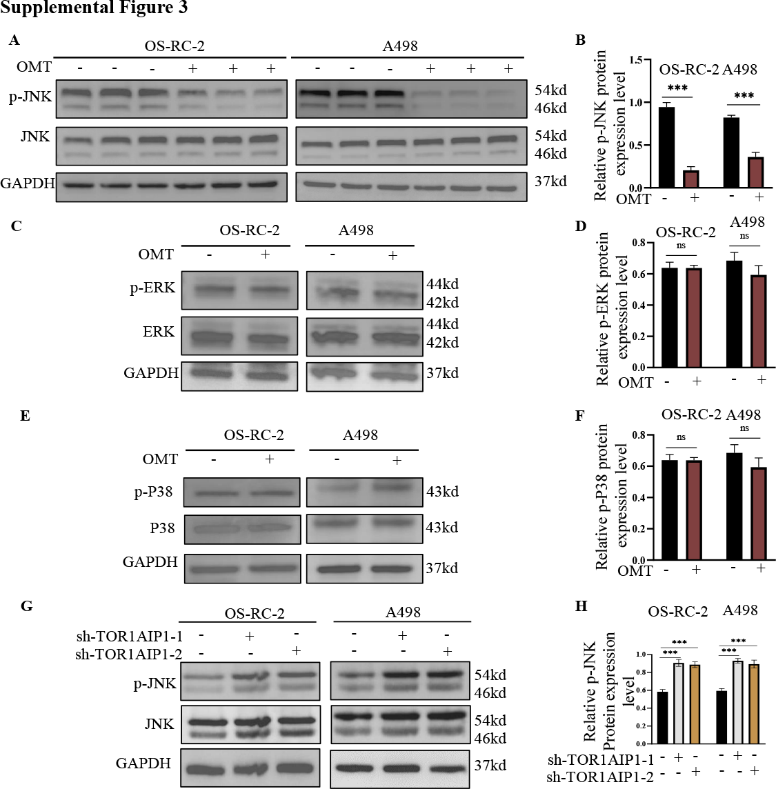


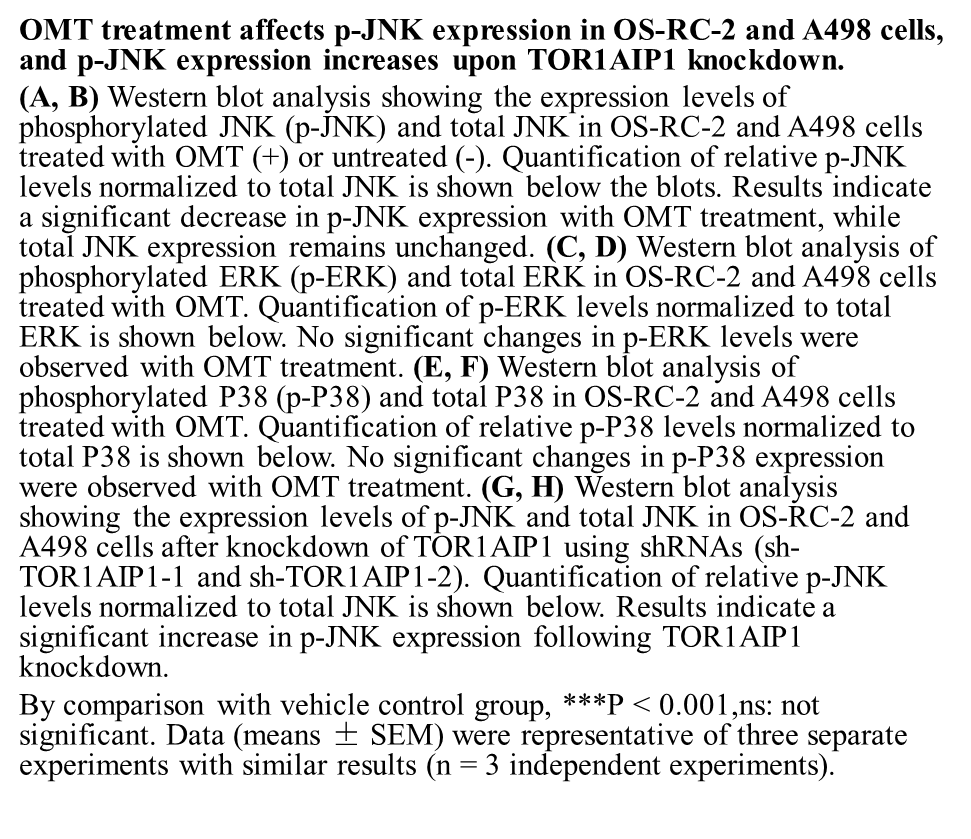


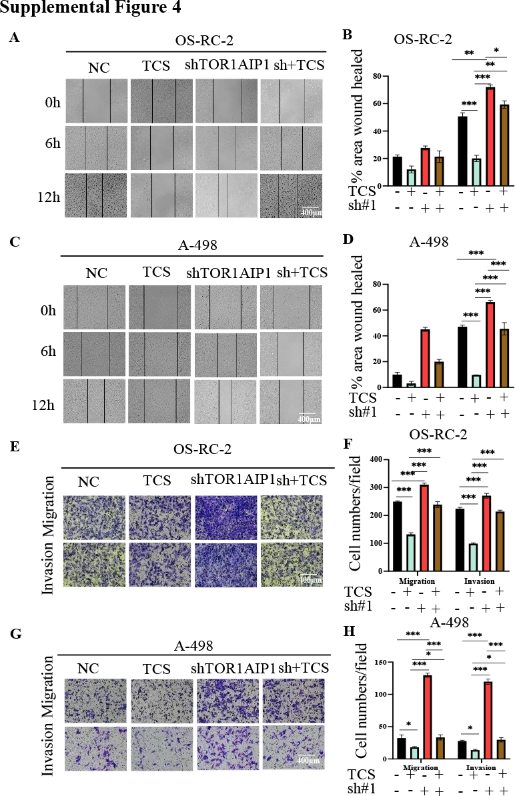


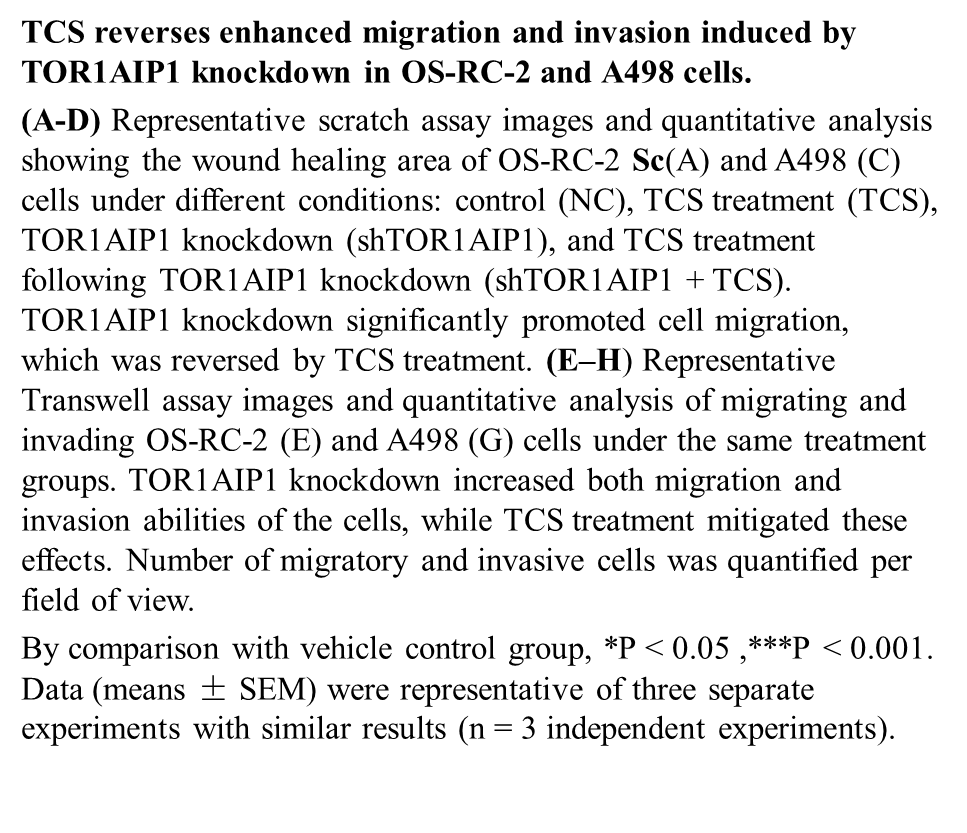


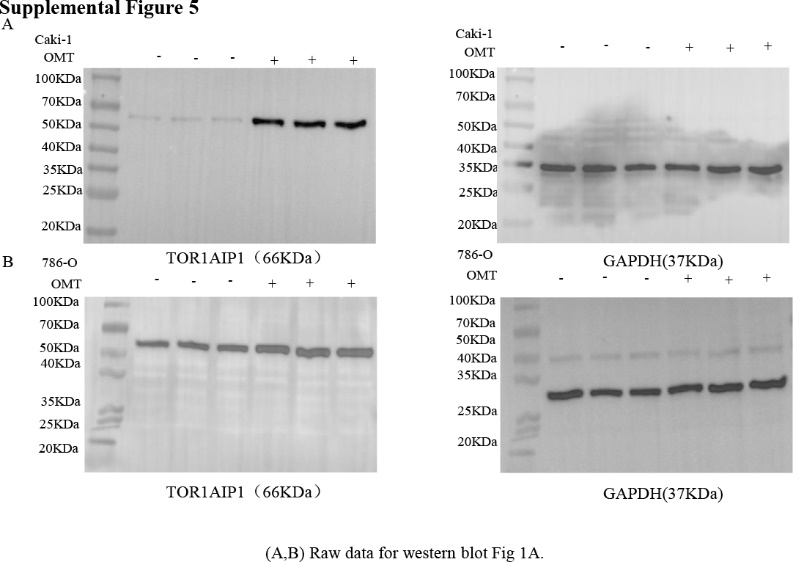


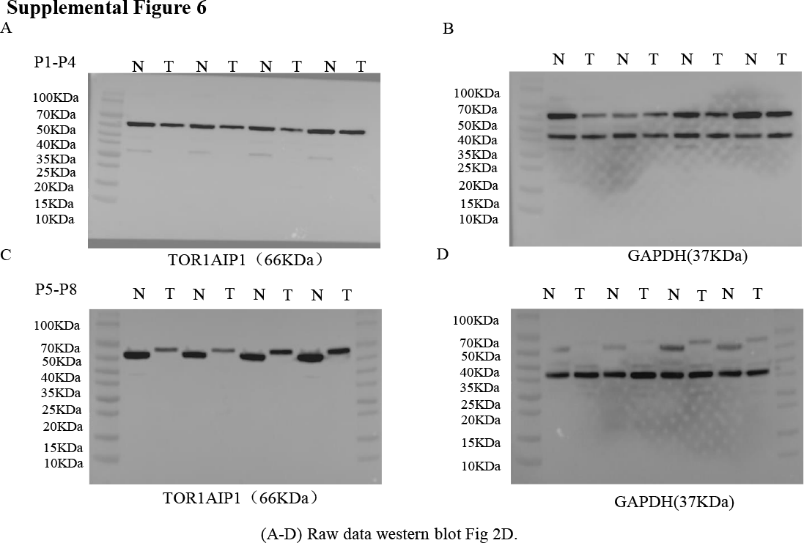


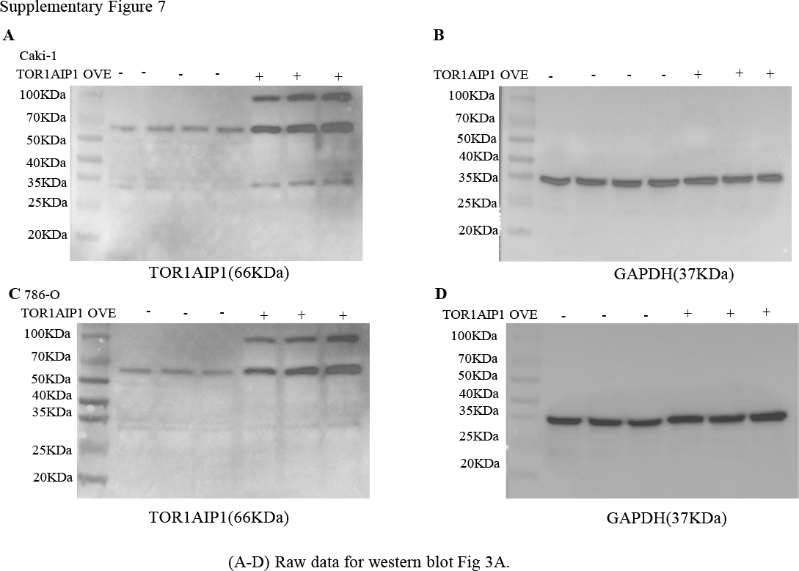


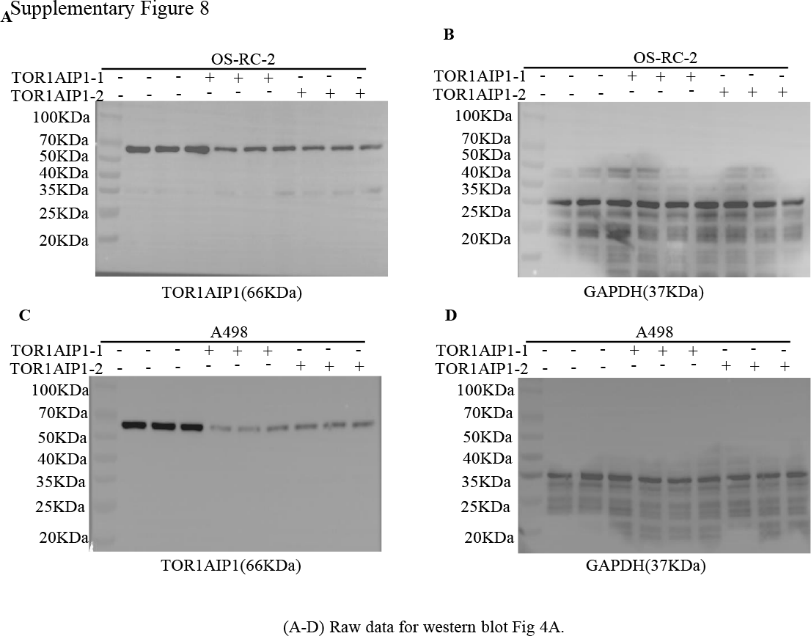


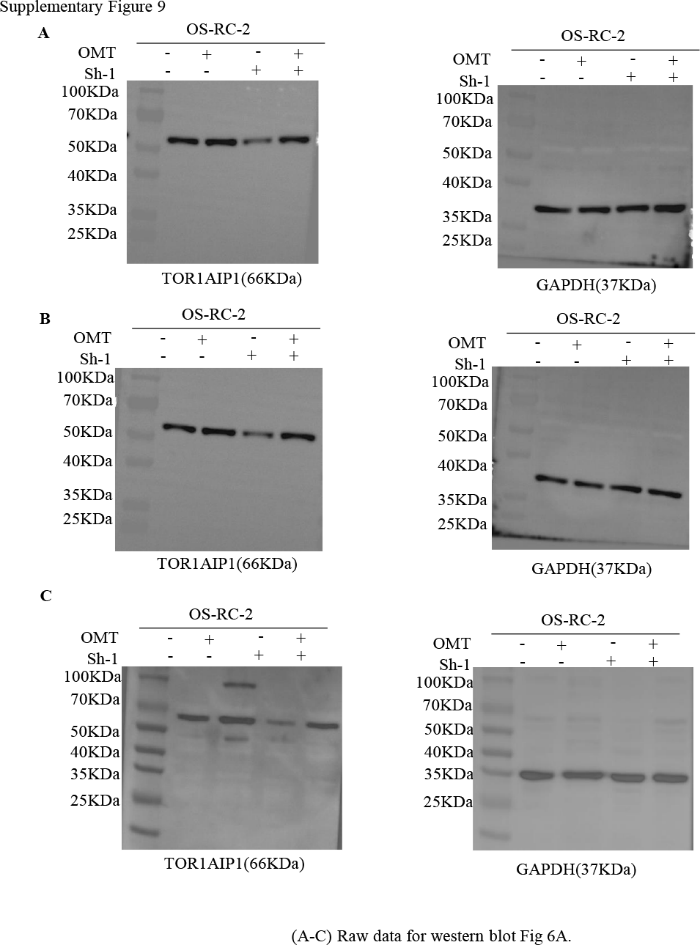


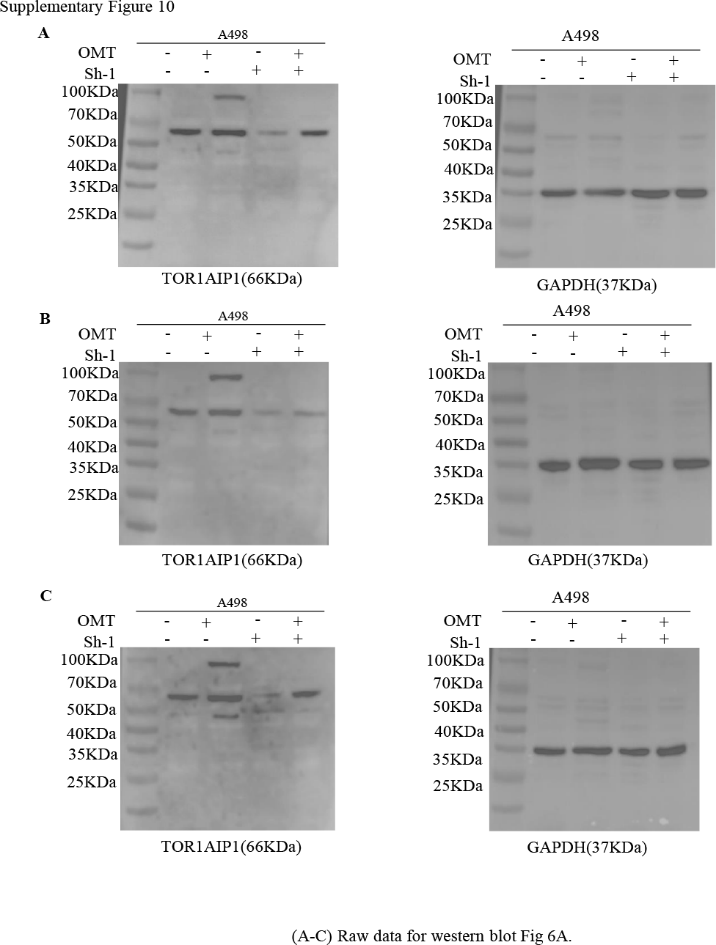


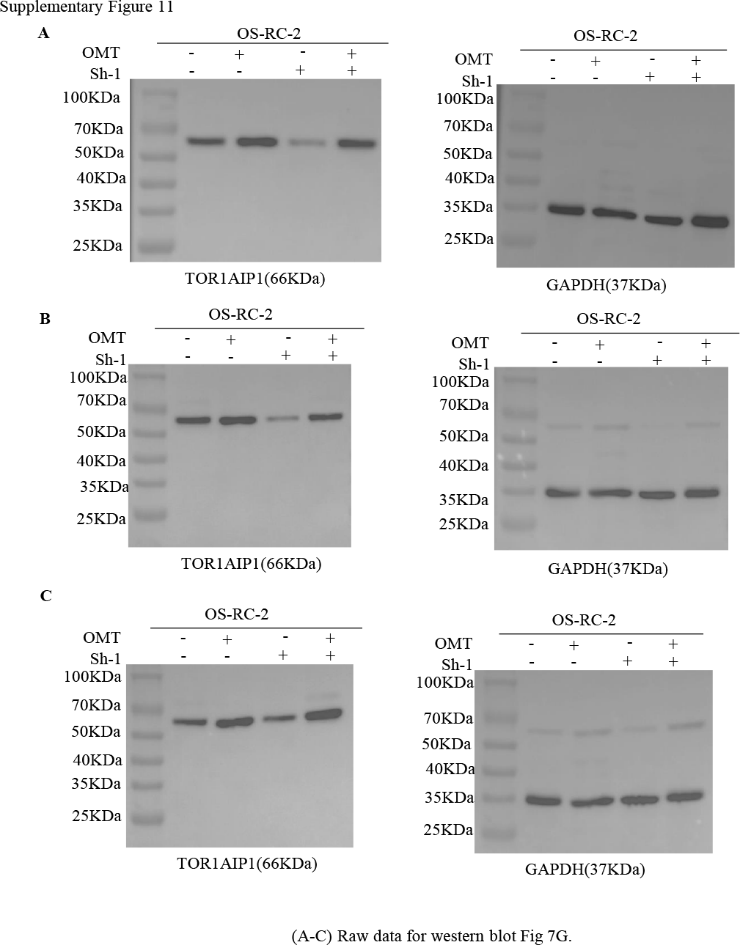


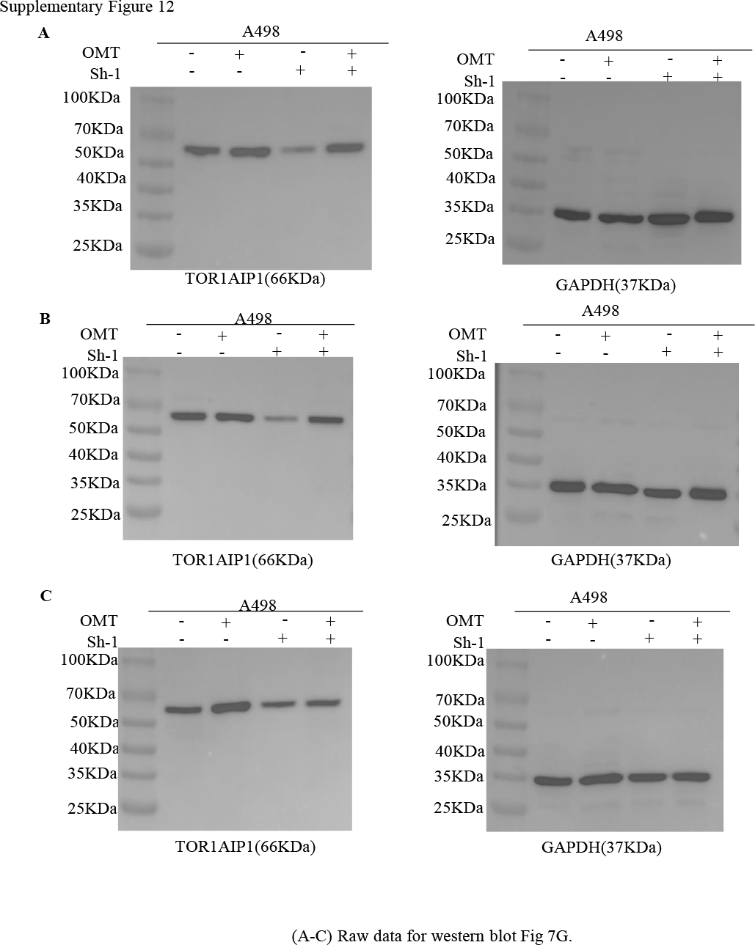


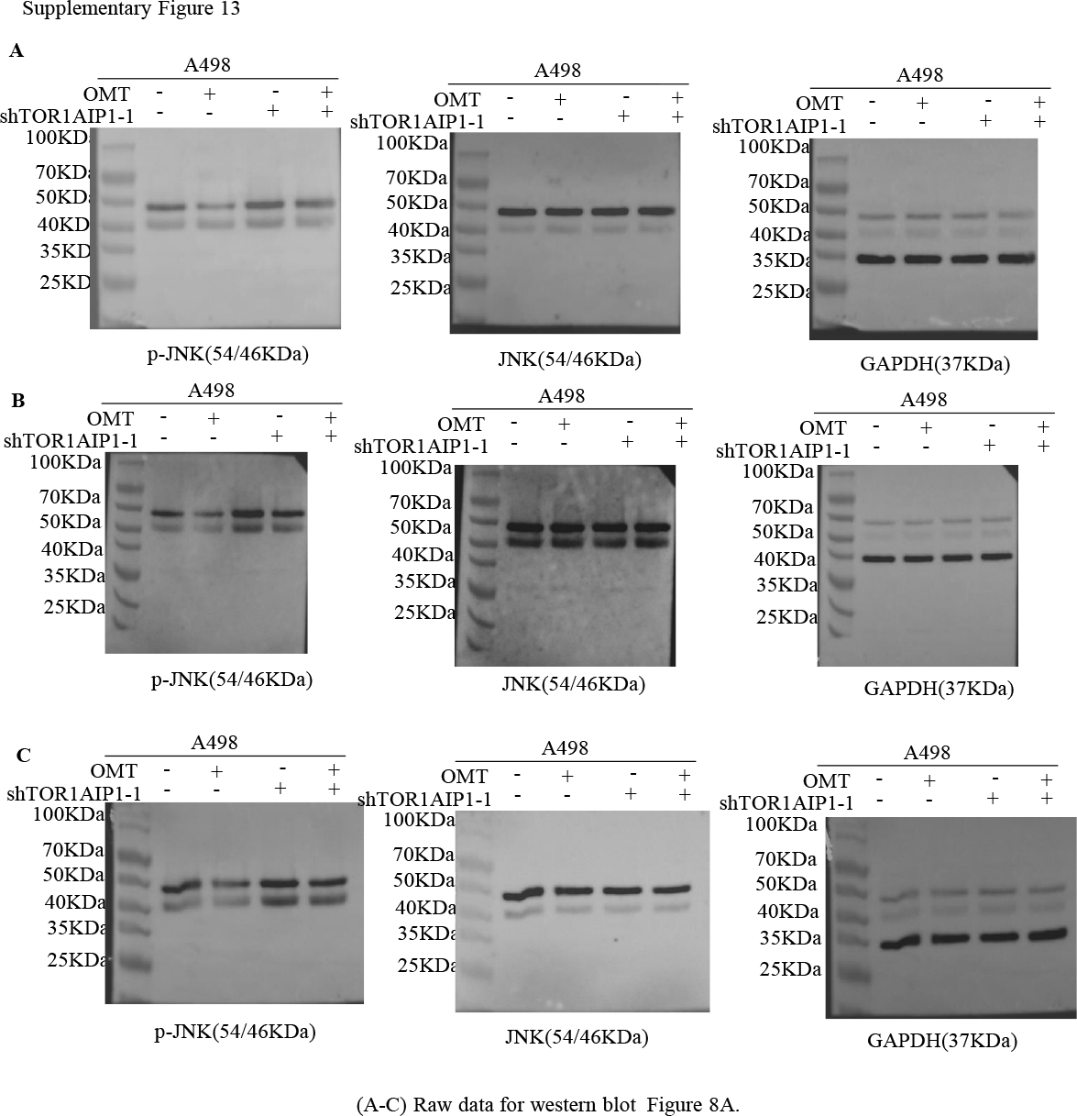


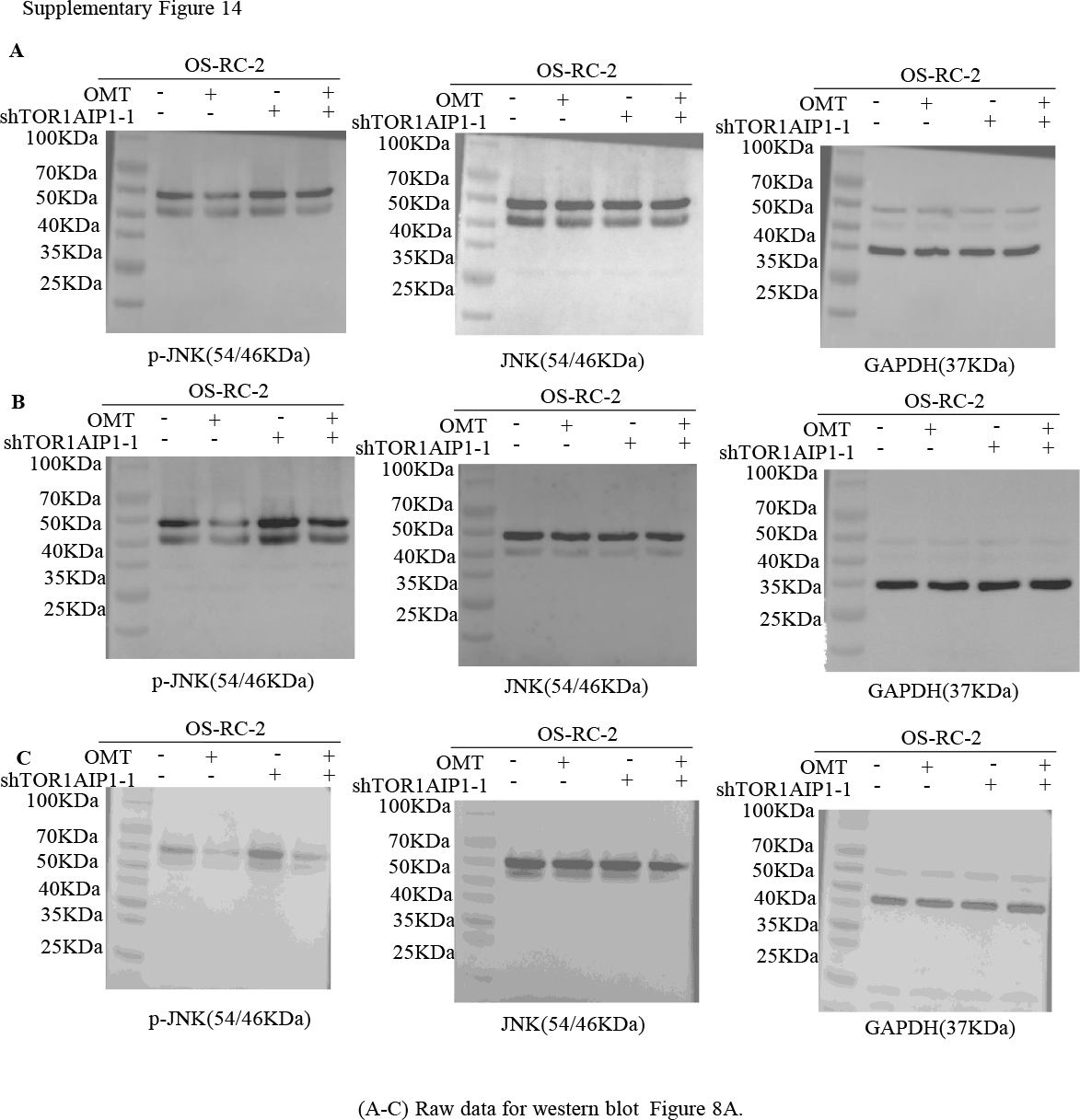


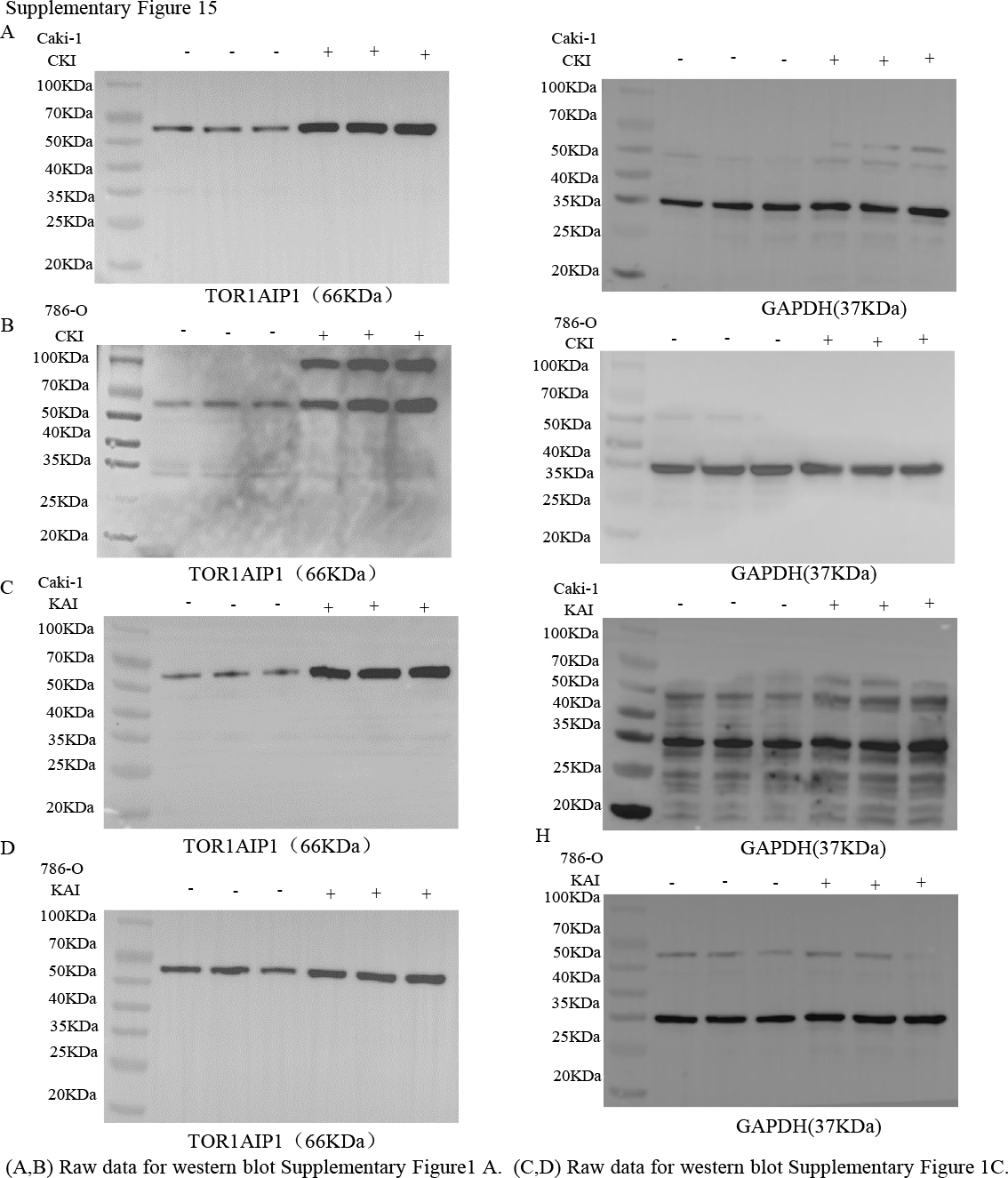


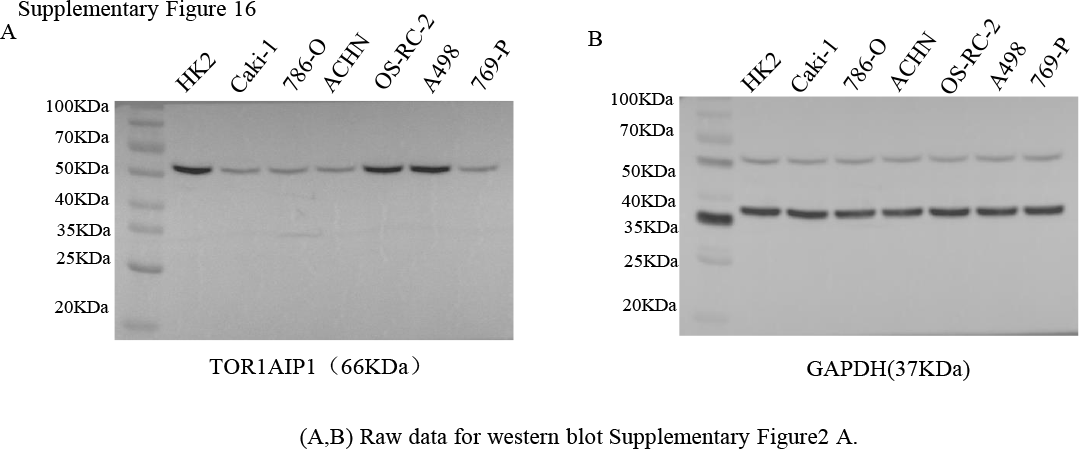


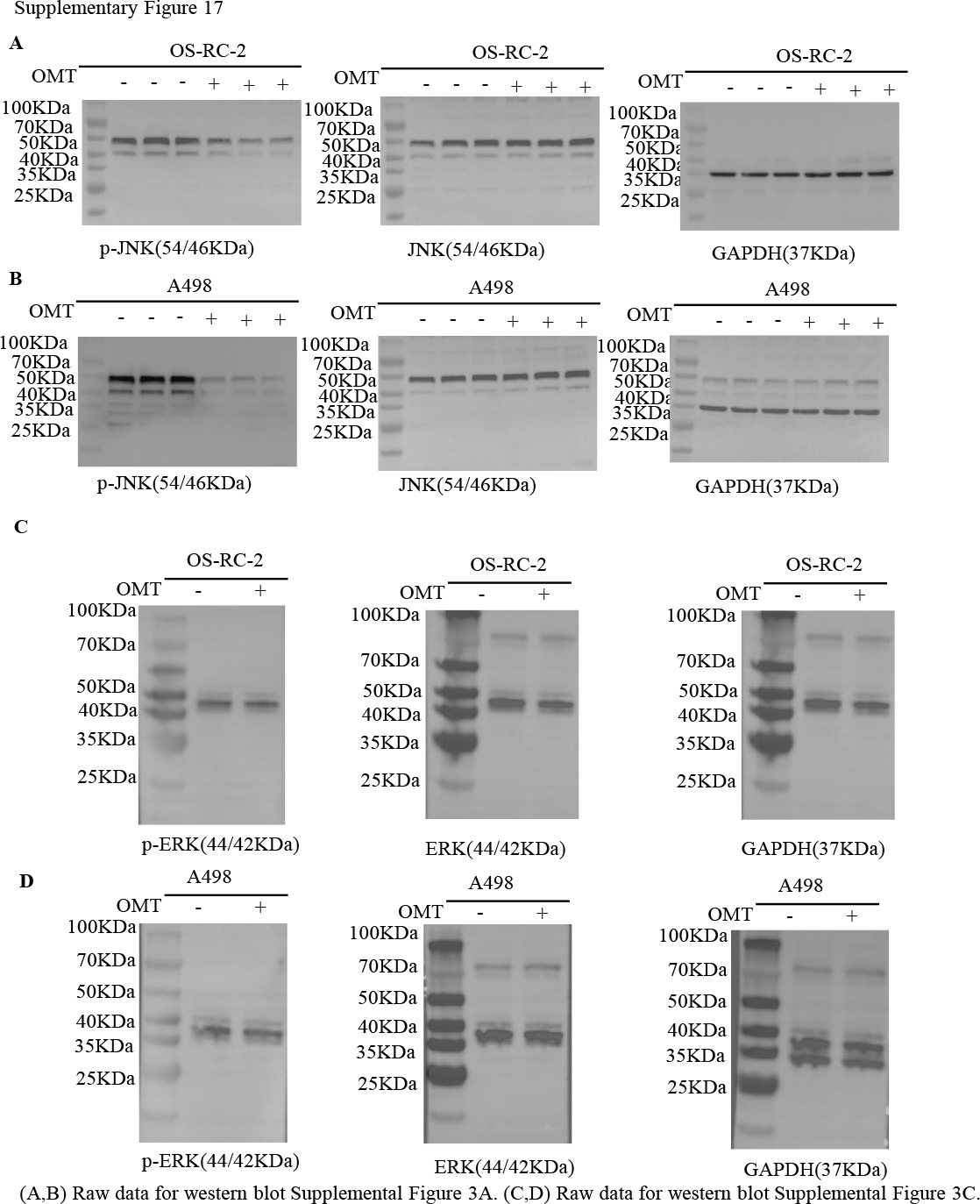


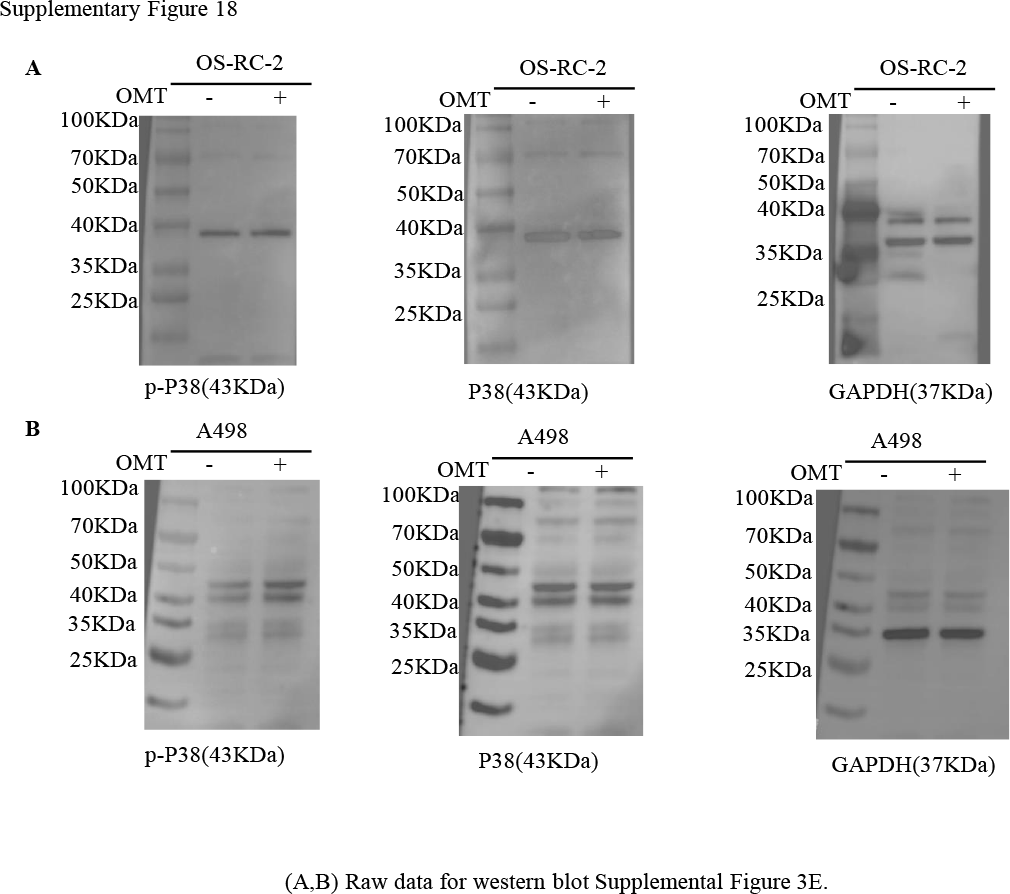


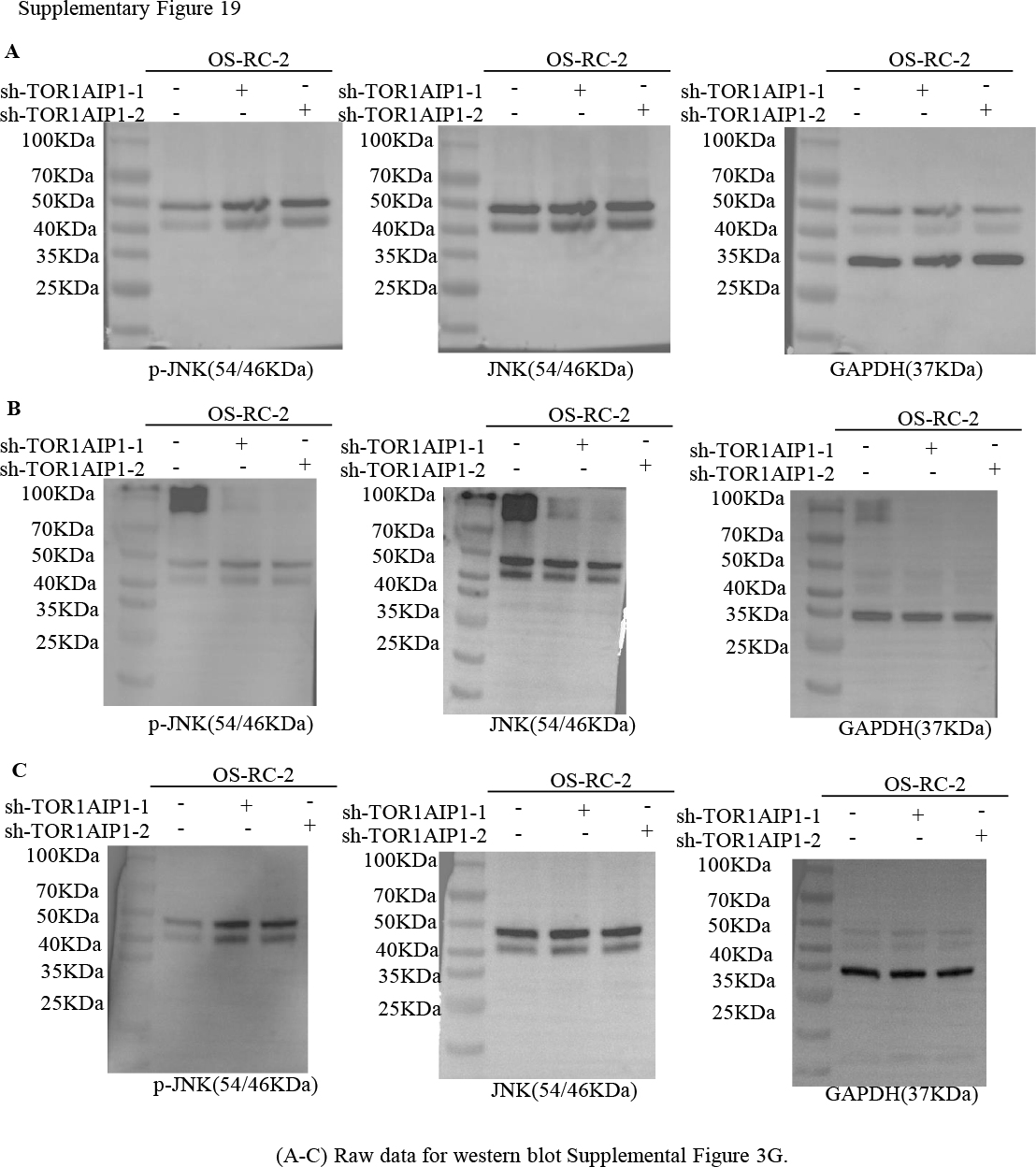


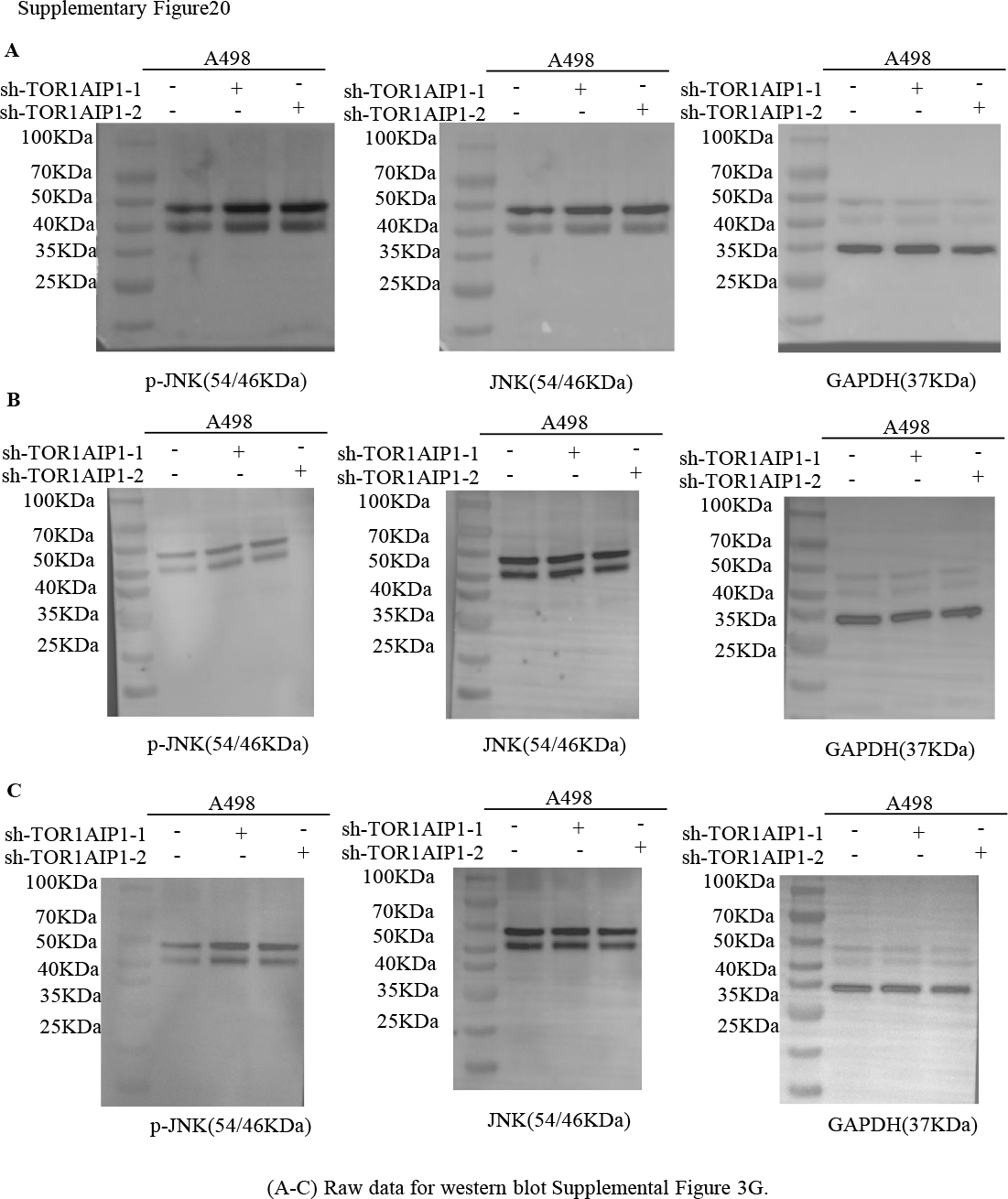

Supplement: Supplementary file 1 [file DataSheet1.docx]
